# Crystal Structures of a Piscine Betanodavirus: Mechanisms of Capsid Assembly and Viral Infection

**DOI:** 10.1371/journal.ppat.1005203

**Published:** 2015-10-22

**Authors:** Nai-Chi Chen, Masato Yoshimura, Hong-Hsiang Guan, Ting-Yu Wang, Yuko Misumi, Chien-Chih Lin, Phimonphan Chuankhayan, Atsushi Nakagawa, Sunney I. Chan, Tomitake Tsukihara, Tzong-Yueh Chen, Chun-Jung Chen

**Affiliations:** 1 Institute of Biotechnology, National Cheng Kung University, Tainan, Taiwan; 2 Life Science Group, Scientific Research Division, National Synchrotron Radiation Research Center, Hsinchu, Taiwan; 3 Institute for Protein Research, Osaka University, Suita, Osaka, Japan; 4 Institute of Chemistry, Academia Sinica, Taipei, Taiwan; 5 Division of Chemistry and Chemical Engineering, California Institute of Technology, Pasadena, California, United State of America; 6 Picobiology Institute, Graduate School of Life Science, University of Hyogo, Kamigori, Hyogo, Japan; 7 Department of Physics, National Tsing Hua University, Hsinchu, Taiwan; 8 Center for Bioscience and Biotechnology, National Cheng Kung University, Tainan, Taiwan; Washington University, UNITED STATES

## Abstract

Betanodaviruses cause massive mortality in marine fish species with viral nervous necrosis. The structure of a *T* = 3 Grouper nervous necrosis virus-like particle (GNNV-LP) is determined by the *ab initio* method with non-crystallographic symmetry averaging at 3.6 Å resolution. Each capsid protein (CP) shows three major domains: (i) the N-terminal arm, an inter-subunit extension at the inner surface; (ii) the shell domain (S-domain), a jelly-roll structure; and (iii) the protrusion domain (P-domain) formed by three-fold trimeric protrusions. In addition, we have determined structures of the *T* = 1 subviral particles (SVPs) of (i) the delta-P-domain mutant (residues 35−217) at 3.1 Å resolution; and (ii) the N-ARM deletion mutant (residues 35−338) at 7 Å resolution; and (iii) the structure of the individual P-domain (residues 214−338) at 1.2 Å resolution. The P-domain reveals a novel DxD motif asymmetrically coordinating two Ca^2+^ ions, and seems to play a prominent role in the calcium-mediated trimerization of the GNNV CPs during the initial capsid assembly process. The flexible N-ARM (N-terminal arginine-rich motif) appears to serve as a molecular switch for *T* = 1 or *T* = 3 assembly. Finally, we find that polyethylene glycol, which is incorporated into the P-domain during the crystallization process, enhances GNNV infection. The present structural studies together with the biological assays enhance our understanding of the role of the P-domain of GNNV in the capsid assembly and viral infection by this betanodavirus.

## Author Summary

Betanodaviruses belong to the family *Nodaviridae* and cause the mortality of numerous larval-stage fish species. Here we report protein crystal structures of a piscine betanodavirus, the Grouper nervous necrosis virus (GNNV), in four different forms. Highlights are two structural features that contribute to the viral molecular mechanisms of the *T* = 3 and *T* = 1 capsid assembly: a calcium-associated protrusion domain and a functional arginine-rich motif. These results also shed insights into the structural basis for evolutionary lineage of the family *Nodaviridae*.

## Introduction

*Nodaviridae* is a family of positive-sense single-stranded RNA viruses with a non-enveloped *T* = 3 capsid. These viruses are characterized by a viral genome comprising two RNA molecules–RNA1 and RNA2. RNA1 (3.1 kb) encodes protein A, which is a RNA-dependent RNA polymerase (RdRp) responsible for viral RNA replication [1,2]. RNA2 (1.4 kb) encodes the structural protein associated with assembly of the viral particle. The subgenomic RNA3, located at the 3’-terminal region of RNA1, encodes a non-structural B2 protein, which plays a role in inhibition of host RNA interference (RNAi) [3–6].

Alphanodaviruses and betanodaviruses are the major genera in the family *Nodaviridae* [7]. Alphanodaviruses infect primarily insects, and are related to the Nodamura virus (NoV; PDB ID: 1NOV), Black beetle virus (BBV; PDB ID: 2BBV), Pariacoto virus (PaV; PDB ID: 1F8V) and Flock house virus (FHV; PDB ID: 4FSJ). Betanodaviruses are also called nervous necrosis viruses (NNV) because they cause an acute syndrome of viral nervous necrosis (VNN) [8]. VNN is a serious syndrome disease causing viral encephalopathy or retinopathy, and is responsible for the high mortality at the larval stage among a wide range of species (warm- and cold-water fishes) or even across species from marine to freshwater fishes in the aquaculture industry [9]. Betanodavirus strains are currently classified into four distinct genotypes based on the genes encoding the viral capsid protein (CP). These include the Striped Jack nervous necrosis virus (SJNNV), Tiger puffer nervous necrosis virus (TPNNV), Red-spotted grouper nervous necrosis virus (RGNNV) and Barfin flounder nervous necrosis virus (BFNNV) [10]. Based on genome organization and on phylogenetic analysis of RNA1 or RNA2, additional clusters of unclassified nodaviruses infecting nematodes, moths, butterflies and prawns have been identified recently [11]. One report identifies two unclassified nodaviruses (shrimp nodavirus), *Macrobrachium rosenbergii* Nodavirus (MrNV) and *Penaeus vannamei* Nodavirus (PvNV), which cause muscle necrosis in prawns [12]. These findings suggest that the family *Nodaviridae* includes not only the known types but also other members with a wide distribution.

In the family *Nodaviridae*, an assemblage of 180 CPs form a *T* = 3 capsid of diameter ~29−35 nm. CP is typically composed of the core jelly-roll topology, forming a face-to-face β-sandwich with two pairs of anti-parallel β-sheets [13]. During assembly of the alphanodavirus particle, self-catalyzed cleavage of the precursor protein α generates proteins β and γ, which are required for structural maturation of the capsid [14]. Protein β forms the canonical eight anti-parallel β-strands with N- and C-termini located inside the virus particle. The highly basic N-terminus of protein β is required to neutralize the encapsidated RNA duplex [15,16]; it also acts as a molecular switch to control the heterogeneous size and shape of the particles [17]. The structural complementarities between the different strains of the genus alphanodavirus appear conserved, despite the existence of large evolutionary distances in phylogenetic relations [7]. However, there is no significant homology in the CP sequences between alphanodaviruses and betanodaviruses. Genotypes of the RGNNV-strain betanodavirus isolated from different grouper species, such as Orange-spotted grouper nervous necrosis virus (OSGNNV), Dragon grouper nervous necrosis virus (DGNNV) and Malabaricus grouper nervous necrosis virus (MGNNV), contain highly conserved genomes. Three uninterrupted major domains of MGNNV CP, including the N-terminal region, the β-sandwich surface domain and the trimeric protrusion domain, have been previously studied by cryo-electron microscopy (cryo-EM) imaging at 23 Å resolution and 3D-PSSM prediction [18]. However, there is currently no high-resolution structural information on the capsid-related organization of the genus betanodavirus.

In this report, we describe the crystal structure of the grouper nervous necrosis virus (GNNV) of the genus betanodavirus in various forms: (i) a complete *T* = 3 GNNV-like particle (GNNV-LP) at 3.6 Å resolution; (ii) *T* = 1 subviral particles (SVPs) of the delta-P-domain mutant at 3.1 Å; (iii) the N-ARM deletion mutant at 7.0 Å; and (iv) the individual P-domain of GNNV CP at 1.2 Å. The crystal structure of GNNV-LP demonstrates several significant and distinct variations in capsid architecture and molecular mechanisms of capsid assembly compared to the genus alphanodavirus and other RNA viruses. In particular, we have identified the conserved structural characteristics of the shell domain on GNNV. Various forms of the *T* = 3 and *T* = 1 GNNV capsids show that the N-terminal arginine-rich motif (N-ARM) acts as a molecular switch. Second, the P-domain, with its DxD motif together with two bound Ca^2+^ ions, plays a pivotal role in the trimerization of the GNNV CP and the particle assembly. These high-resolution structural details contribute further to our in-depth understanding of the molecular mechanisms of viral assembly and infection, and should provide the structural basis for studying the evolution of the family *Nodaviridae*.

## Results

### *T* = 3 icosahedral structure of GNNV-LP

**Fig 1:**
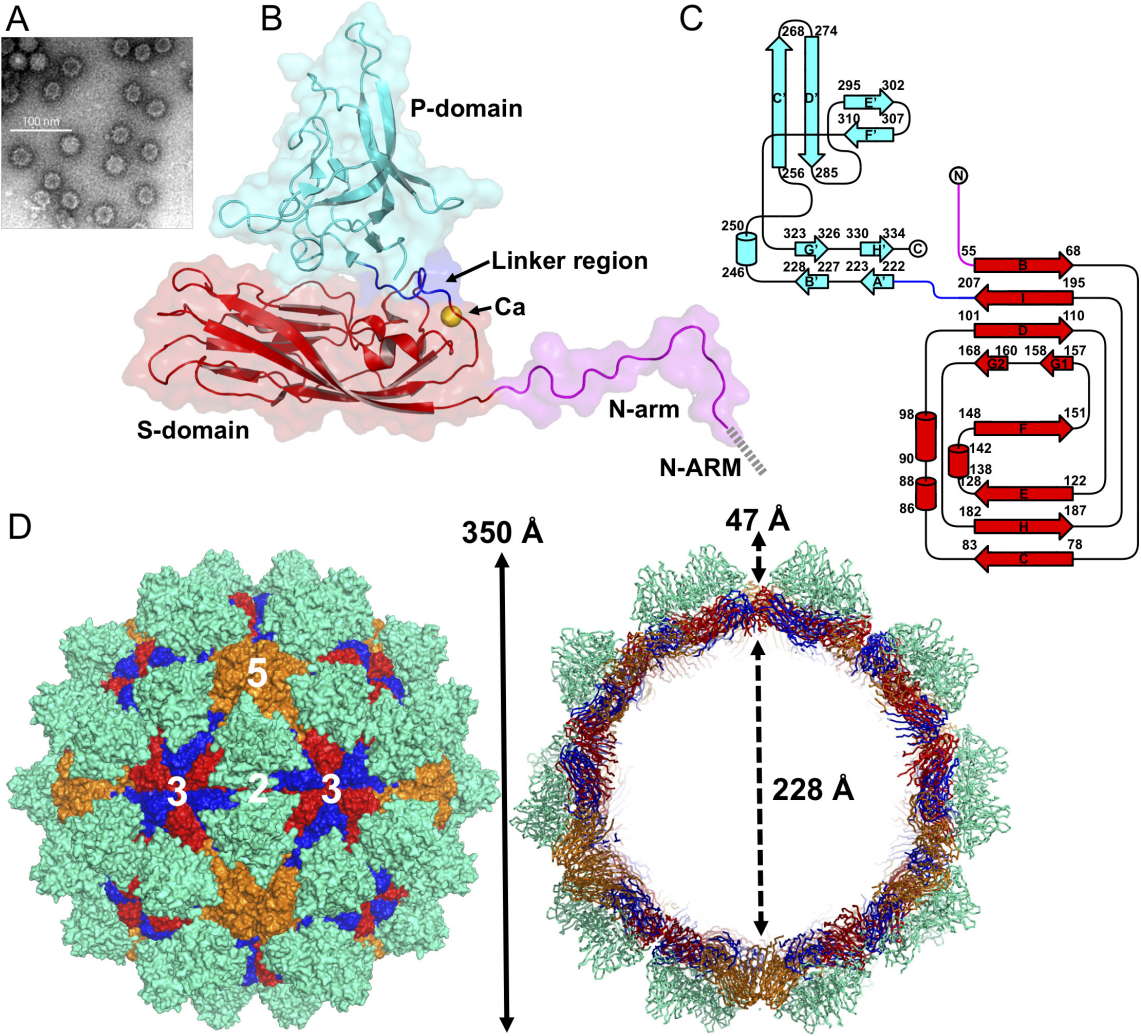
EM image and the overall structure of GNNV-LP. (A) A representative negative-staining EM image of the purified GNNV-LPs after self-assembly. (B) A ribbon presentation of the subunit C of GNNV-LP. The disordered N-ARM (residue 1−33, gray), N-arm (residues 34−51, magenta), the S-domain (residues 52−213, red), the linker region (residues 214−220, blue), the P-domain (residues 221−338, cyan) and Ca^2+^ ion (yellow sphere) are shown. (C) A topology diagram of GNNV CP with the helices and strands in cylinders and arrows, respectively. The 1D topology of the subunit C is color-coded as in *B*. (D) Surface domain-colored diagram (*left*) and central cavity (*right*) representations of the *T* = 3 GNNV-LP. The tip-to-tip distance is ~350 Å, the diameter of the central cavity is ~228 Å, and the spike protrusion on the capsid surface is ~47 Å. The S-domains of the subunits A, B and C are shown in orange, blue and red, respectively, and the P-domains are shown in cyan. The structure of the GNNV-LP is viewed along the I2, I3 and I5 axes.

SUMO-GNNV CPs are overexpressed in *Escherichia coli* (*E*. *coli*) and the GNNV-LPs are self-assembled *in vitro*. Based on the EM images, the morphology of GNNV-LP shows a *T* = 3 capsid with a diameter of 30~35 nm (Fig 1A and S2A Fig). We determine the crystal structure of the *T* = 3 GNNV-LP using the *ab initio* method with non-crystallographic symmetry (NCS) averaging and refine the structure to 3.6 Å (S1 Fig). The electron density of the icosahedral asymmetric unit (iASU) of the *T* = 3 GNNV-LP allows modeling of residues 52−338 for subunits A and B, and residues 34−338 for subunit C. The rest of the N-terminal segment of each subunit, which contains N-ARM, the positively charged arginine-rich motif _23_RRRANNRRRSN_33_, is disordered.

The overall topological structure of the GNNV CP consists of the N-terminal arm (N-arm) (residues 34−51), the shell domain (S-domain) (residues 52−213), the linker region (residues 214−220) and the protrusion domain (P-domain) (residues 221−338) (Fig 1B). The ordered N-arm exists along the icosahedral two-fold (I2) interface of the inner surface, and extends its N-terminus to the icosahedral three-fold (I3) axis to form a β-annulus. The S-domain comprises an eight-stranded anti-parallel β-sandwich with three short α-helices, which is a canonical structural feature similar to other virus CPs [13]. The individual S- and P-domains of the GNNV CP, connected by the flexible linker region, do not interact with each other directly. The P-domain folds into an independent structure, including eight anti-parallel β-strands and a short α-helix connected with loops of various lengths (Fig 1C).

Sixty trimeric S-domains participate in inter-subunit contacts, forming a continuous thin shell of the capsid with an empty inner cavity. Three neighboring P-domains per iASU embrace one another at the *quasi* three-fold (Q3) axes to form 60 protrusions on the particle surface (Fig 1D). Three neighboring monomeric S-domains from subunits A, B and C are engaged in dimeric, trimeric and pentameric interactions along the I2, I3 and icosahedral five-fold (I5) axes (Fig 1D). Although the GNNV CP (338 residues) is shorter than the alphanodavirus CP (407 residues), the structural organization of the GNNV capsid with its 60 large protrusions reveals a *T* = 3 architecture with a particle size similar to the compact alphanodavirus structure, in which the N- and C-termini of the CP are both positioned within the capsid.

### Structural characterization of the N-terminus of the CP

**Fig 2:**
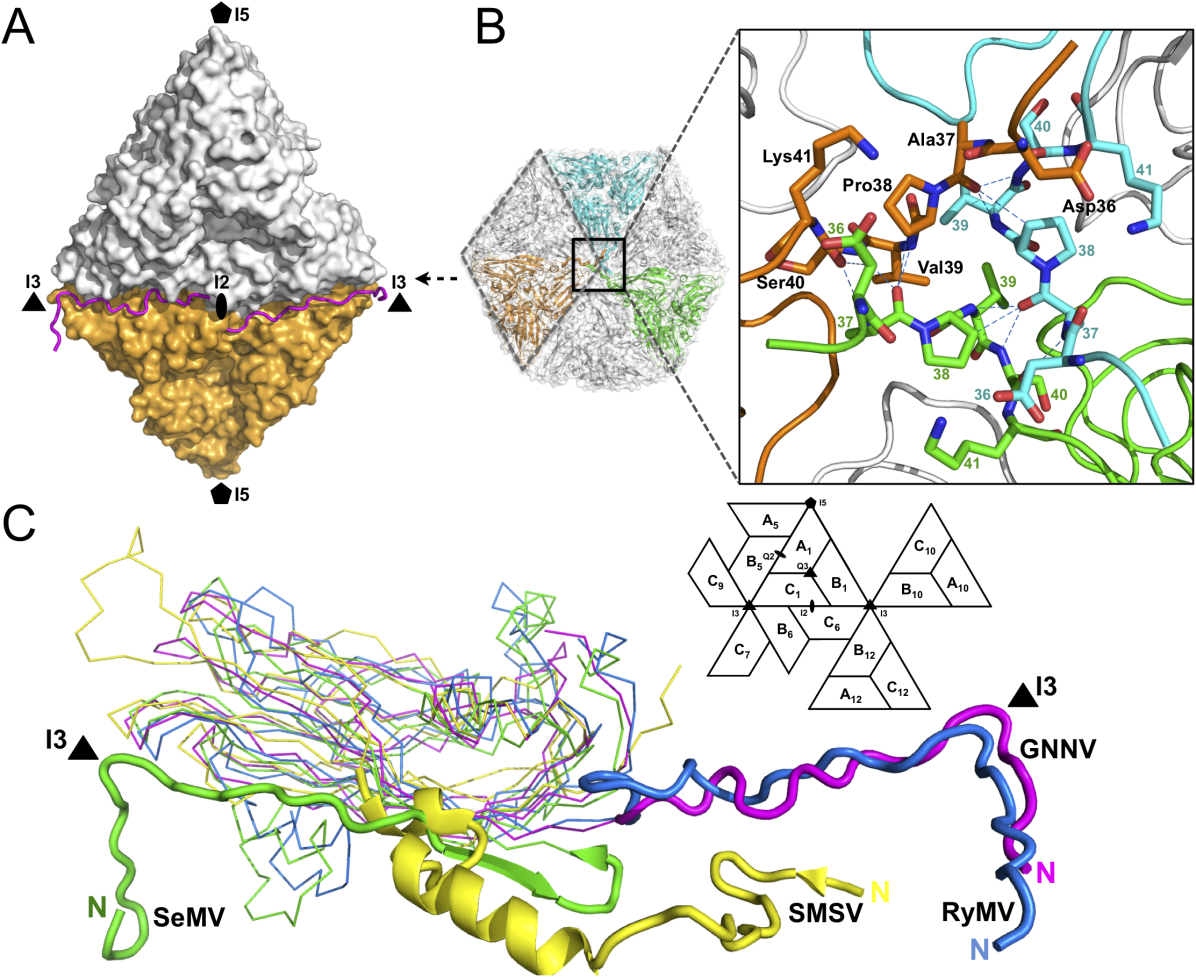
Structural organizations of the N-arm and the β-annulus. (A) Two N-arms from the subunit-C/C dimer along the I2 interface. N-arms are colored in magenta. Two iASUs are shown in white and orange. (B) A representation of the hexameric capsomers and the β-annulus as viewed along the I3 axis from the inner surface. Three iASUs are shown in cyan, green and orange, respectively (*left*). Residues 36−41 of three different C-subunits form a β-annulus structure as an enlarged central section (*right*). The hydrogen bonds are shown with blue dotted lines. (C) A comparison of the N-arms with an alignment of the S-domains (subunit C) from four different representative viruses: SeMV (green), SMSV (yellow), RyMV (blue) and GNNV (cyan) with a nomenclature diagram. The orientations of the N-arm between GNNV and SeMV are towards opposite different I3 axes (black triangle).

Only the partial N-terminus of each subunit C is seen inside the capsid; the N-termini of subunits A and B are completely absent. The first 33 residues of the N-termini, namely the N-ARM, are disordered in all the subunits. This flexible structural feature of the basic N-ARM is thought to play an important role in the RNA encapsidation in the intact virus. Two ordered and extended N-arms from the subunit-C/C dimer, together with their corresponding N-ARMs, occupy the groove of the inner surface along the I2 interface (Fig 2A). Residues 36−41 from subunits C_1_, C_10_ and C_12_ are engaged through hydrogen bonding to form a β-annulus structure around the I3 axis (Fig 2B). The β-annulus structure of GNNV is similar to that of the Rice yellow mottle virus (RyMV) [19], but differs from that of the Sesbania mosaic virus (SeMV), in which three N-arms from subunits C_1_, C_7_ and C_9_ form a β-annulus structure around another I3 axis [20,21]. Notably, each genotype of the genus betanodavirus has a conserved residue, Pro38, for stabilization of the β-annulus structure, and this proline residue corresponds to Pro35 in RyMV and Pro53 in SeMV (Fig 2B) [19–21]. The N-arm of subunit C_1_ in GNNV is oriented at the B_1_-C_6_ interface toward one I3 axis, similar to that in RyMV. In contrast, the N-arm of subunit C_1_ in *T* = 3 RNA plant viruses, such as SeMV, folds back to result in an anti-parallel topology facing the first β-strand B of the S-domain. This results in a hairpin conformation along the I2 interface. The San Miguel sea lion virus (SMSV) of the family *Caliciviridae* also contains three ordered N-arms from the C_1_, C_10_ and C_12_ subunits located near the I3 axis similar to GNNV, but oriented toward another direction (Fig 2C) [22]. Thus, based on structural conformation, the N-arms of the viral CP can be classified into several categories.

### The icosahedral scaffold of the S-domain with Ca^2+^ ions incorporation

The S-domains of the CPs in the GNNV-LP form a conserved jelly-roll structure as in those of canonical viruses [13]. Within each CP, two four-stranded anti-parallel sheets (β-strands BIDG and CHEF) are connected with two α-helices between strands C and D and one α-helix between strands E and F, respectively. A search of structural homologs between the GNNV S-domain and the corresponding domain in the CPs of other viruses using the *DALI* program [23] shows the highest similarity with the Orsay virus (Z-score 23.8) [24] and the Carnation mottle virus (CMV) (Z-score 18.1) [25].

The CP subunits adapt to the *quasi*-equivalent interactions of the triangulated icosahedral lattices, suggesting that the N-terminus of the CP is a molecular switch to adjust the curvature of the subunit-A/B dimer along the *quasi* two-fold (Q2) axis and the subunit-C/C dimer along the I2 axis during *T* = 3 particle assembly [26]. The bent conformation of the subunit-A/B dimer in GNNV-LP is similar to that observed for the CP in the alphanodavirus. The flat conformation of the subunit-C/C dimer is stabilized by two ordered N-arms alone, in contrast to alphanodavirus, where incorporation of the encapsidated RNA participates in the *T* = 3 quaternary organization [15,16]. The strand B and the D-E loop on subunit C interact with the N-arm from the neighboring subunit C_6_ through hydrogen bonds to stabilize the subunit-C/C dimer.

Divalent metal ions, such as calcium, are typically associated with metal-coordinating residues for particle formation, stability and infectivity [27]. The GNNV-LP has three Ca^2+^ ions located at interfaces between pairs of subunits within each of the S-domains, which are coordinated with side chains of Asp130 and Asp133 to form the _130_ **D**xx**D**xD_135_ motif at the E-F loop, Gln100 at the C-D loop, Ser170 at the G-H loop and Glu213 near the linker region from the neighboring subunit (S4A Fig). There are three S-domains per iASU, and they all share the same calcium-binding structures to facilitate subunit-subunit interactions, similar to those seen in the CP of some RNA plant viruses, such as tombusvirus (**D**x**D**xxD) [28–30] and SeMV (**D**xx**D)** [31]. In contrast, alphanodavirus utilizes Asp249 and Glu251 to form **D**x**E**xxD motif and incorporate one or two Ca^2+^ along the Q3 axis in its CP [27,32].

The electrostatic potential surface in the region of the S-domain of the GNNV-LP shows distributions of positively- and negatively-charged regions that are more dispersed on the inner surface compared with *T* = 3 PaV (S4B Fig). Earlier crystal and cryo-EM structures of the PaV have suggested that 30 copies of an ordered encapsidated RNA duplex formed a dodecahedral cage within the inner surface [15] These data indicate that the encapsidated RNA of GNNV may be involved in a non-specific interaction with the inner surface or a specific interaction with positively charged residues of the flexible N-ARM inside the GNNV capsid.

### The overall structure of the trimeric P-domains

In the cryo-EM structure of MGNNV, 60 large protrusions along the Q3 axes have been identified that are larger than the extended domain (34 residues) of alphanodavirus [18]. Our crystal structure of the *T* = 3 GNNV-LP also shows 60 protrusions on the particle surface along the Q3 axes formed by three contiguous P-domains per iASU. Although the structure of the P-domain can be readily assigned, the protrusions of the GNNV-LP show too poor electron density after NCS-averaging to allow a complete characterization of the morphology of the P-domain, which might be caused by the high flexibility.

**Fig 3:**
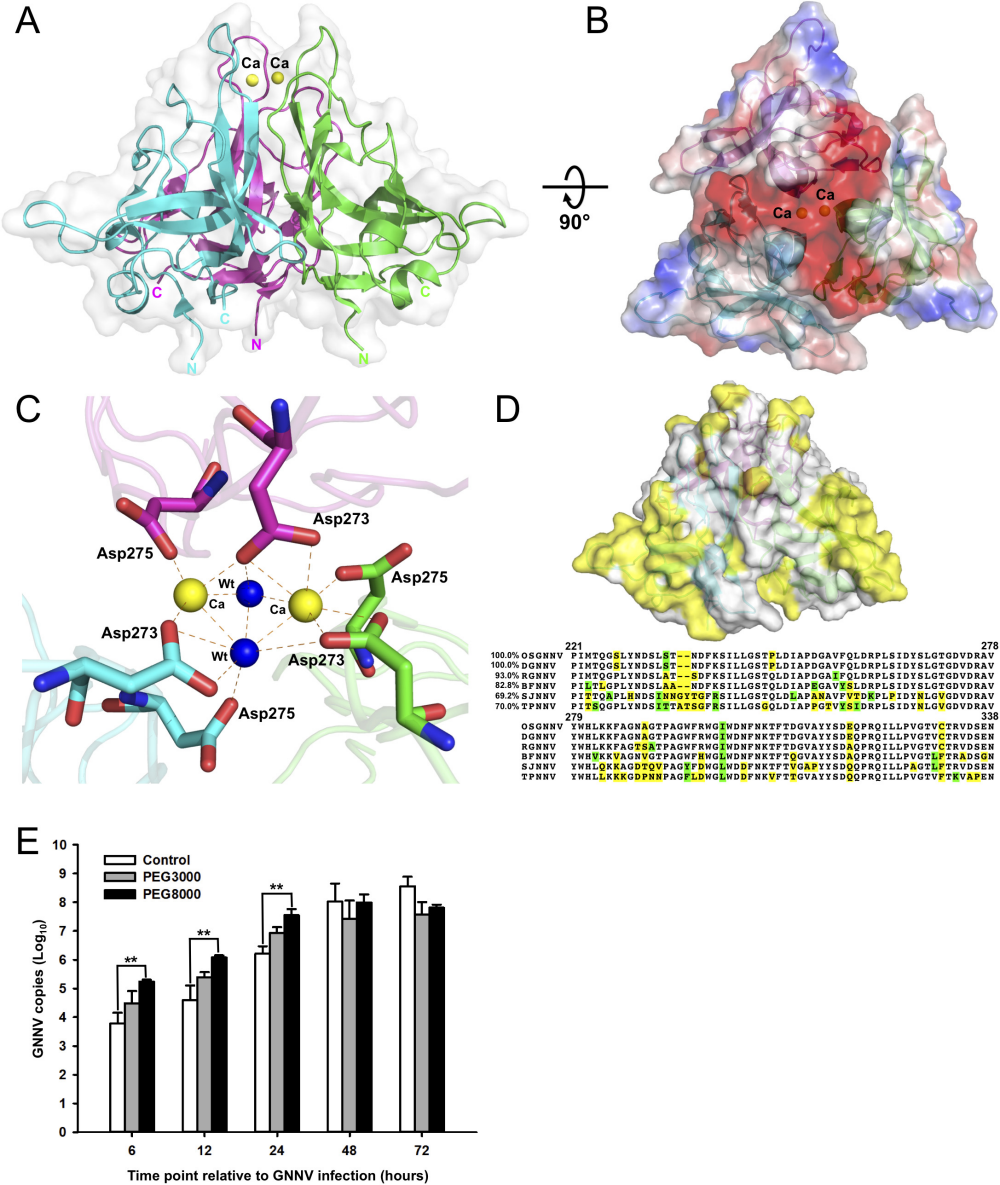
Trimeric interactions of the P-domain with Ca^2+^ ions and the functional role of PEG during GNNV infection. (A) The ribbon model of trimeric P-domains. The structure of trimeric P-domains (magenta, green and cyan) is shown with two Ca^2+^ ions (yellow spheres) bound at the top region. (B) The electrostatic surface potential of the trimeric P-domains. A top view of the trimeric P-domains is rotated 90° along the horizontal axis from *A*, and colored in red and blue for negative and positive charges viewed along the three-fold axis. (C) The calcium-binding region of the trimeric P-domains. Two Ca^2+^ ions (yellow spheres) and two water molecules (blue) are coordinated with three sets of _273_ **D**x**D**_275_ motifs (sticks) from neighboring subunits colored in magenta, green and cyan, respectively. The hydrogen bonds are shown with orange dotted lines. (D) Sequence-alignment variables mapped onto the surface of the P-domain from different genotypes of betanodavirus. The hypervariable regions (yellow) from OSGNNV, DGNNV, RGNNV, BFNNV, SJNNV and TPNNV are represented on the surface of the trimeric P-domains (*upper*). The comparison of representative T4 genomic regions of the P-domains of different genotypes of betanodavirus is shown (*lower*). Strictly variants and similar residues are colored in yellow (as in D, *upper*) and green, respectively. (E) Improvement of GNNV infection by PEG treatment. PEG8000 assists GNNV infection in GF-1 cells. Intracellular GNNV RNA2 copies in GF-1 cells were determined by real-time qPCR in log scales after GNNV infection at the indicated time. Data are represented as mean ± SD of three independent experiments and analyzed by one-way ANOVA test, *P < 0.05; **P < 0.01; ***P < 0.001.

To gain more complete and detailed structural information, we have determined the crystal structure of the truncated P-domain (residues 214−338) at high resolution (1.2 Å; Fig 3A and S7A Fig). Since it lacks only the S-domain, crystal packing of the truncated P-domain remains a trimer in the ASU, with a similar orientation and formation as the P-domains in the GNNV-LP. The anti-parallel β-strands C’ and D’ are located at the interface of the Q3 axis, presenting an L-shaped geometry facing the other six-stranded β-sheets (A’, B’, E’, F’, G’ and H’). This conformational arrangement is different from the jelly-roll topology. The flexible C-terminus of the P-domain is located near the linker region at the interface space between the S- and P-domains in the GNNV-LP. Structural alignments of the GNNV P-domain with all structures in the PDB reveal low degrees of structural similarities with the P-domain of Orsay virus (Z-score 5.1) [24], the P1-domain of Hepatitis E virus (HEV) (Z score 2.2) [33,34] and the P1-domain of Calicivirus (Z-score 2.1) (S5 Fig) [22].

### Calcium ions, water molecules and amino-acid variations on the P-domain

The high-resolution structure of the truncated P-domain has allowed us to clearly locate two Ca^2+^ ions near the non-crystallographic three-fold axis, which are coordinated with the C’-D’ loop to stabilize the trimeric structural fold (Fig 3A and 3B and S7A Fig). The _273_ **D**x**D**_275_ motif on the C’-D’ loop from each neighboring subunit interacts with two Ca^2+^ ions and two water molecules through electrostatic and hydrogen-bonding interactions. This calcium-binding site is buried in the cavity of the protrusion at a distance of ~37 Å from the S-domain (Fig 3A and 3B). The distances between two Ca^2+^ ions and the side chains of Asp273 and Asp275 from each subunit are *ca*. 2.4~2.5 Å. Notably, only two of the three Asp275 residues are asymmetrically coordinated to the two Ca^2+^ ions, and the other Asp275 coordinates with one water molecule. A similar asymmetrical binding of two Ca^2+^ ions and two water molecules with three Asp273 is observed (Fig 3C). Analysis of the elution profiles of the P-domain after size-exclusion chromatography (SEC) showed a possible role of Ca^2+^ in the trimerization of P-domains, suggesting that formation of the trimeric structure of the P-domains might be initiated and completed in the absence and presence of Ca^2+^, respectively (S6B Fig).

Water molecules have been observed at the inter-subunit interfaces within the complete viral capsid; they must be important in stabilizing association of the subunits [35]. From the high-resolution structure, we have delineated the distribution of water molecules in the P-domains. As mentioned above, there are two water molecules at the calcium-binding site providing the trimeric contacts and stabilizing the protrusion (Fig 3C). At the interface between the D’ and E’ strands with the F’-G’ loop from neighboring subunits, we also find two invariant water molecules associated with _278_VYWH_281_, Gly299, Gln322 and Ile323 through hydrogen bonds, which are also essential to maintain the conformation and stability of each of the trimeric P-domains (S6A Fig).

A multiple amino-acid sequence alignment of P-domains from different genotypes of the genus betanodavirus reveals that several regions, including residues 223−227, 233−237, 253−259 and 285−291, are divergent. Notably, all these residue variations are located on the surface of the protrusion in the structure of the truncated P-domain (Fig 3D).

### Enhancement of GNNV infection by polyethylene glycol

An inspection of the structure of the truncated P-domains reveals additional electron densities in several pockets on the surface. We consistently find three glycerol (GOL) molecules located between the B’-C’ and F’-G’ loops of the three P-domains, and one polyethylene glycol (PEG) molecule at the interface between the two F’-G’ loops from neighboring subunits (S7B Fig). The *B*-factor values of GOL and PEG molecules are 21.6 and 26.5 Å^2^, respectively. A previous study has showed that PEG could increase the ability of Hepatitis B virus (HBV) to bind to the cell surface and to enhance virus infection [36]. On the basis of this lead, we examine the grouper fin cell line (GF-1) infected with GNNV in the presence (4%) of PEG3000 or PEG8000, respectively. Compared to the untreated group, the viral copy number was significantly higher in the presence of PEG, especially PEG8000 (~30 folds), within 24 hours (Fig 3E). These data suggest that the infectivity of GNNV for GF-1 cells could be enhanced with PEG8000 (4%) during infection. Based on the PEG-binding ability of the P-domain, we surmise that the presence of PEG might participate in the early step(s) of GNNV infection.

### Particle polymorphism and subunit organization in *T* = 3 and *T* = 1 GNNV assembly

The symmetry of the icosahedral particles can be related to a regulation process that dictates the choices of inter-subunit arrangements or protein-nucleotide interactions to guide the capsid assembly [37]. For *T* = 3 GNNV-LP, the N-terminus of the CP contains the disordered N-ARM for putative RNA interactions at the inner cavity of the particles. The next N-arm is ordered along the I2 interface only on subunit C. The P-domains of GNNV-LP show an independent trimeric organization, which is different from that of the S-domains. We therefore speculate that the N-ARM or P-domain of GNNV-LP might act as a major molecular switch in regulating *T* = 3 or *T* = 1 assembly. To address this issue, we have constructed two sub-clones, including (i) the delta-P-domain mutant (residues 35−217) and (ii) the N-ARM deletion mutant (residues 35−338), and have determined their structures (Table 1).

**Table 1:** Data collection, phase extension and refinement statistics.

	*T* = 3 GNNV-LP (4WIZ)	Truncated P-domain (214−338) (4RFU)	*T* = 1 delta-P-domain mutant (35−217) (4RFT)	*T* = 1 N-ARM deletion mutant (35−338)
**Data collection**				
Wavelength (Å)	0.900	1.000	1.000	0.900
Temperature (K)	100	100	100	100
Space group	*C*2	*P*2_1_2_1_2_1_	*P*4_2_	*P*6_3_22
Cell dimensions				
*a*, *b*, *c* (Å)	477.4, 422.7, 337.9	65.0, 83.5, 85.6	289.0, 289.0, 175.1	260.8, 260.8, 250.5
*α*, *β*, *γ* (°)	90.0, 90.0, 134.0	90.0, 90.0, 90.0	90.0, 90.0, 90.0	90.0, 90.0, 120.0
Resolution (Å)	266.0−3.6	30.0−1.2	30.0−3.1	30.0−7.0
	(3.66−3.6)	(1.24−1.2)	(3.21−3.1)	(7.25−7.0)
*R*_merge_ ^†^ *	0.198 (0.795)	0.044 (0.388)	0.191 (0.814)	0.152 (0.665)
*R*_p.i.m._ ^*¶*^ *	0.105 (0.587)	0.020 (0.271)	0.097 (0.433)	0.075 (0.328)
*I/*σ_*I*_ *	7.7 (1.5)	31.3 (2.5)	8.3 (1.8)	12.3 (3.2)
Completeness (%)*	99.4 (92.0)	99.2 (95.1)	98.3 (96.8)	100.0 (100.0)
Redundancy*	4.0 (2.7)	5.4 (2.7)	4.7 (4.2)	4.8 (4.9)
**Phase extension (266−3.1 Å)**				
Averaging *R* factor	0.276	−	−	−
Correlation coefficient	0.885	−	−	−
**Refinement Refinement**				
*R*_cryst_ ^‡^/*R*_free_ ^§^ (%)	25.5 / 29.7	17.5 / 18.1	23.8 / 28.8	−
No. of atoms				
Protein	203160	2739	75180	−
Ligand	−	31	−	−
Calcium	90	2	−	−
Water	−	542	−	−
*B*-factors (Å^2^)				
Protein	103.78	13.76	54.4	−
Ligand	−	22.76	−	−
Calcium	38.14	8.06	−	−
Water	−	23.56	−	−
R.m.s deviations				
Bond lengths (Å)	0.016	0.008	0.010	−
Bond angles (°)	1.767	1.044	1.590	−

*Values in parentheses are for highest-resolution shell.

^†^ *R*_sym_ = ∑_*hkl*_∑_*i*_|*I*_*i*_(*hkl*) − 〈*I*(*hkl*)〉|/∑_*hkl*_∑_*i*_ *I*_*i*_(*hkl*), where *I*_*i*_(*hkl*) is the *i*th measurement and 〈*I*(*hkl*)〉 is the weighted mean of all measurements of *I*(*hkl*).

^*¶*^ *R*_p.i.m._ = ∑_*hkl*_{1/[*N*(*hkl*) − 1]}^1/2^∑_*i*_|*I*_*i*_(*hkl*) − 〈*I*(*hkl*)〉|/∑_*hkl*_∑_*i*_ *I*_*i*_(*hkl*).

^‡^ *R*_cryst_ = ∑_*hkl*_|*F*_*o*_ − *F*_*c*_|/∑_*hkl*_ *F*_*o*_, where *F*_*o*_ and *F*_*c*_ are the observed and calculated structure factor amplitudes of reflection *hkl*.

^§^ *R*_free_ is as *R*_cryst_, but calculated with 5% of randomly chosen reflections omitted from refinement

**Fig 4:**
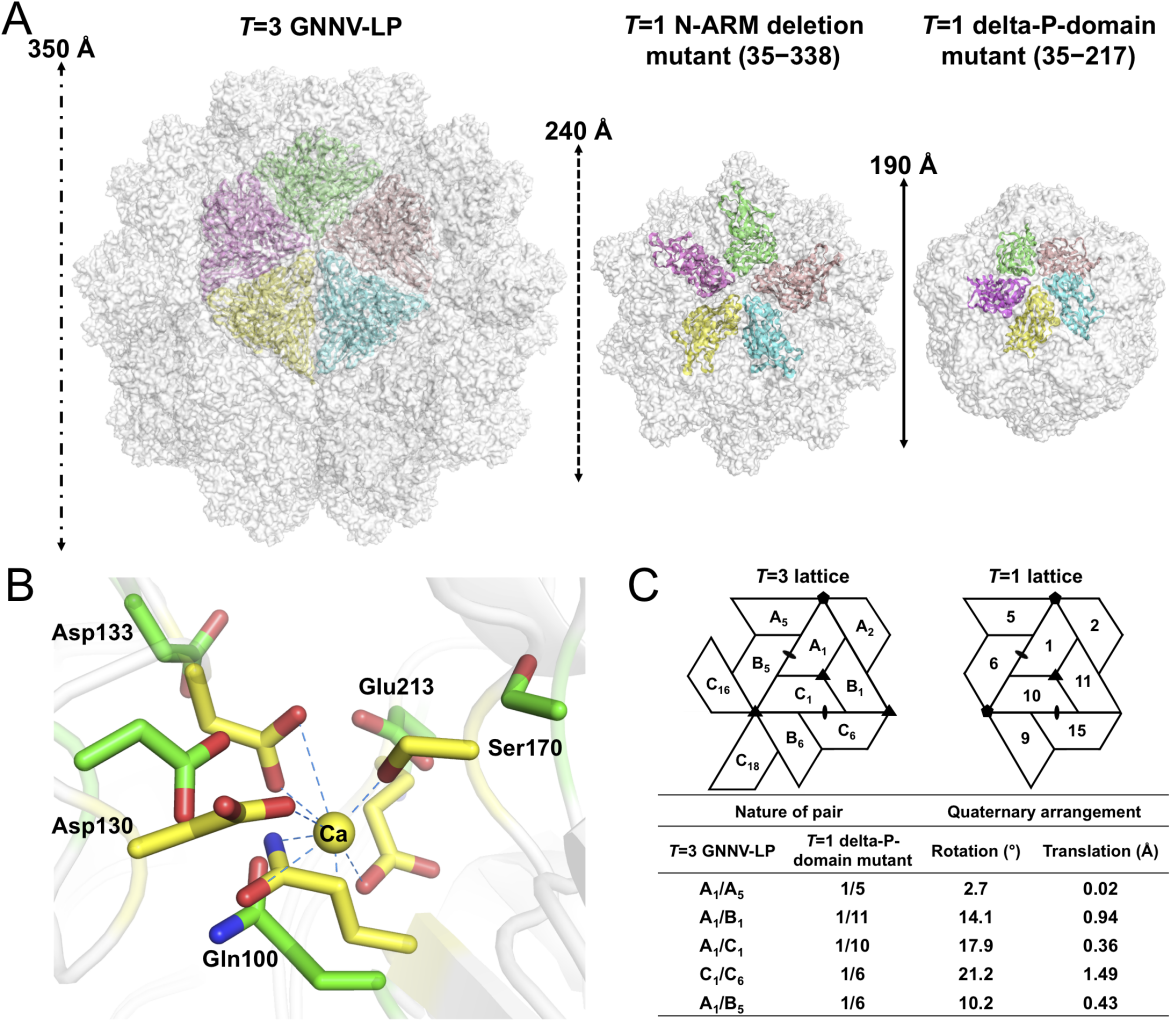
*T* = 1 SVPs of GNNV. (A) Surface presentations of the monomers engaged in pentameric interactions in the *T* = 3 and *T* = 1 GNNV capsids. Each iASU is viewed along the I5 axis and shown in green, wheat, cyan, yellow and magenta colors, respectively. The diameters of three capsids are indicated. (B) A superimposition of the calcium-binding regions of the S-domain between the *T* = 3 GNNV-LP and the *T* = 1 delta-P-domain mutant. Gln100, Asp130, Asp133, Ser170 and Glu213, which participate in the Ca^2+^ coordination in the *T* = 3 GNNV-LP (yellow), exhibit conformational changes in the *T* = 1 delta-P-domain mutant (green). The hydrogen bonds are indicated with blue dotted lines. (C) A comparison of the spatial relationship of subunit pairs in the *T* = 3 GNNV-LP and the *T* = 1 delta-P-domain mutant. Subunit packing and the nomenclature of the *T* = 3 and the *T* = 1 capsids are shown. Rotation angles (°) and translation distances (Å) are identified and compared between different subunit pairs of the *T* = 3 and the *T* = 1 GNNV capsids.

In the delta-P-domain mutant, sixty copies of the S-domain assemble with interactions of I2, I3 and I5 symmetries into a *T* = 1 SVP with a diameter ~190 Å (Fig 4A and S2C Fig). Only residues 52−214 of each subunit are observed at a resolution of 3.1 Å. As expected, the delta-P-domain mutant comprises a canonical eight-stranded anti-parallel β-sandwich with three short α-helices similar to the S-domain of *T* = 3 GNNV-LP. In the N-ARM deletion mutant, the crystals diffract to only 7 Å resolution. However, analyses of self-rotation functions and molecular replacement indicate that the N-ARM deletion mutant could form *T* = 1 capsid of diameter ~240 Å, which is consistent with the EM images (Fig 4A and S2B and S3 Figs).

Although the organization of the equivalent subunits around the I3 axes of the *T* = 1 delta-P-domain mutant is notably similar to the arrangement of the iASU subunits of the *T* = 3 GNNV-LP, the organization of the trimeric subunits is flatter than that of the *T* = 3 GNNV-LP. In the *T* = 1 delta-P-domain mutant, there is no Ca^2+^ observed at corresponding calcium-binding sites as seen in the S-domain of *T* = 3 GNNV-LP. The hollow or empty binding site exhibits an expanded geometry with maximum movement of ~2.6 Å of the main chains (Fig 4B).

We compare the quaternary organizations of the *T* = 1 delta-P-domain mutant and the *T* = 3 GNNV-LP by superimposing dimeric, trimeric and pentameric partners in order to evaluate the rotation and translation of selected subunit-pairs. The differences in rotational angles and translations at the interfaces of several subunits are identified (Fig 4C). We find that, without the Ca^2+^-mediated interactions at the subunit interfaces, the weaker contacts cause changes in the inter-subunit organization, with expanded assembly of the *T* = 1 delta-P-domain mutant, similar to the structure in the Asp mutants of *T* = 1 SeMV [38].

## Discussion

**Fig 5:**
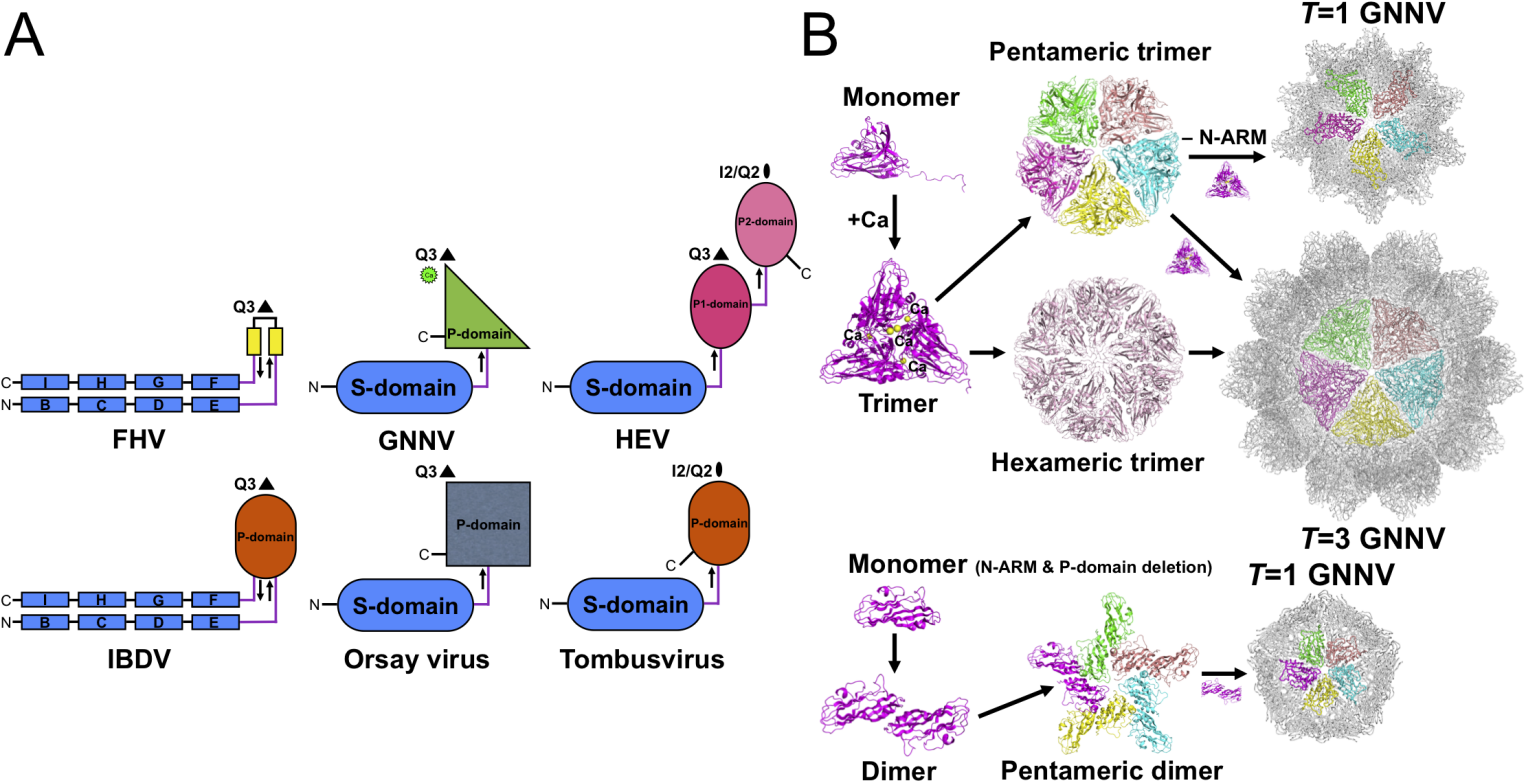
Evolutionary lineages and self-assembly mechanisms of GNNV. (A) Structural characteristics of different categories of RNA viruses. Simplified topology cartoons (β-strand, rectangles; jelly-roll structure, capsules; β-barrel; ovals) of CPs from different viruses, including FHV, IBDV, GNNV, Orsay virus, HEV and tombusvirus are shown. S-domains are colored in blue; P-domains are colored in smudge, gray and orange, respectively; the P1- and P2-domains are indicated in magenta and pink, respectively. The Ca^2+^ ion in the P-domain of GNNV is labeled as a green sphere. The direction (from N- to C-terminal) of the linker region of each CP is indicated with arrows. (B) Diagram showing the putative self-assembly process of the *T* = 3 and the *T* = 1 GNNV capsids. The P-domain and Ca^2+^ ions (yellow spheres) encode information that controls trimerization of the GNNV CPs for the putative pentameric and hexameric trimers. The N-ARM guides the assembly of the complete *T* = 3 capsid.

In the family *Nodaviridae*, RNA2 encodes the CP required for particle assembly and involved in host specificity. The phylogenetic tree from pairs of matched amino-acid sequences of representative CPs of the family *Nodaviridae* indicates that alphanodavirus and betanodavirus originated in different lineages and were segregated into a significant, distinct hallmark of ancestries [7]. Pairwise evolutionary distances of CPs between different genotypes of betanodavirus are shown to be shorter than those of alphanodavirus (S8A Fig). Our four crystal structures of betanodavirus GNNV, including the complete *T* = 3 GNNV-LP, the truncated P-domain and two *T* = 1 GNNV SVPs, reveal distinct structural conformations and characteristics in the mechanisms of particle assembly. Our data suggest that GNNV can be utilized as a model to understand all other genotypes of betanodavirus in terms of structural and molecular biology (S8B Fig). For instance, the Orsay virus, a yet unclassified agent that infects nematodes, has a CP with a distinct phylogenetic clade in the family *Nodaviridae* that shows some topological similarities with the GNNV CP (Fig 5A and S8A Fig) [24].

### Structural insights into the β-annulus and the N-arm in GNNV

Studies investigating the formation of the β-annulus structure with three conserved proline residues around the I3 axis suggest that the N-arm of GNNV, containing only 18 residues, is too short to cooperate with the first β-strand B of the S-domain to form a hairpin structure as in SeMV [20,21]. Instead, the β-annulus in GNNV is formed by three N-arms from the C_1_, C_10_ and C_12_ subunits at the different I3 axis, with a symmetric geometry similar to RyMV (Fig 2C) [19]. The residues Asp36–Lys41 of the GNNV N-arm with the conserved proline residue (Pro38) contribute to the formation of the β-annulus around the I3 axis via hydrogen bonding, similar to those of other RNA plant viruses, such as RyMV [19], SeMV [20,21], CMV [28], Tomato bushy stunt virus (TBSV) [29,30] and Southern bean mosaic virus (SBMV) [39,40]. Through this comparison, we might infer that the three ordered N-arms contributed by the C-subunits are involved in the trimeric β-annulus structure regardless of the sequence variations of CPs with large evolutionary distances or different fold-classifications of the following N-arms between GNNV and other RNA plant viruses. Furthermore, the flat contacts of the subunit-C/C dimer seem able to create a spacious locus to accommodate two ordered N-arms, which are stabilized by hydrogen bonds, in the *T* = 3 GNNV-LP structure without RNA encapsidation (S4B Fig). This structure feature is different from that in alphanodaviruses such as PaV, where a piece of genomic RNA is incorporated with the ordered arm of the subunit A. This subunit A-RNA interaction has been proposed to be necessary to promote the flat conformation of the subunit-C/C dimer [16]. Taken together, it appears that the formation of the β-annulus with the three conserved Pro38 around the I3 axis, the specific length of N-arm along the I2 interface, and the cavity space created by flat contacts of the subunit-C/C dimer might be essential for the morphology and the order of the N-terminus of CPs during the *T* = 3 GNNV assembly.

### Ca^2+^ binding and cysteine residues in the S-domain

Despite significant variations in amino-acid sequences, the structure of the GNNV S-domain exhibits a jelly-roll topology, similar to other structural viral CPs (Fig 2C). Divalent metal ions, such as Ca^2+^ or Zn^2+^, have been shown to play a crucial role in subunit interactions, particle stability, virion infection and environmental resistance in polyomavirus [41], rotavirus [42], tombusvirus [28–30], sobemovirus [19,31,40] and nodavirus [27,32]. In GNNV, three Ca^2+^ ions per iASU are incorporated into the _130_ **D**xx**D**xD_135_ motif at the interfaces of the S-domains, as found in similar structural regions of *T* = 3 RNA plant viruses [19,28–31,40,43]. Therefore, the characteristic folds of viral CPs might be most likely a consequence of the geometric requirements of the building block, which is favorable as the jelly-roll β-barrel fold with conserved sequence patterns, including a calcium-binding site for the distinctive viral shell architecture [13,44]. Investigation of the Asp mutations on DGNNV [43] and our GNNV-LP structure shows that Asp130 and Asp133, but not Asp135, coordinate with Ca^2+^ for particle formation and stabilization. There are four cysteine residues on the GNNV CP (Cys115, Cys187, Cys201 and Cys331). Based on the failure of VLP formation in the presence of single mutations of either C115A or C201A, the existence of a disulfide-bond linkage between Cys115 and Cys201 was previously postulated [45]. However, a structural inspection of the S-domain of GNNV shows that the distribution of Cys115, Cys187 and Cys201 is too remote to establish intra- or inter-subunit disulfide-bond linkages, implying that the disulfide bond is not required for the proper assembly of the GNNV capsid. The locations of Cys115 and Cys201 in GNNV are similar to those of Cys131 and Cys252 of SeMV [46] but different from those of the Cys105−Cys197 disulfide found in Orsay virus [24] (S4C Fig).

### Topologies of the S-, P-domains and linker region on betanodavirus

Viral CP is generally divided into several categories according to the number of short connecting linkers between the P- and S-domains. We find that the number of linkers, one or two, might correspond to localizations of the N- and C-termini on the opposite or the same side, respectively. The structure of GNNV provides an example of a unique topology with only one linker connecting the P- and S-domains of *T* = 3 GNNV and trimeric P-domains with Ca^2+^ for 60 protrusions along the Q3 axes (Fig 5A). In contrast, the dimeric P-domains for 30 protrusions along the I2 axes and 60 protrusions along the Q2 axes appear with one flexible hinge between P- and S-domains in several *T* = 3 viral capsids, such as the families of *Caliciviridae* and *Tombusviridae* [22,28]. Two anti-parallel linkers with the trimeric P-domains along the Q3 axes have been reported in the infectious bursal disease virus (IBDV) of the family *Birnaviridae*, which is similar to alphanodavirus [47]. There are two independent linkers from the P1-domain around the three-fold axes connecting the S-domain and the P2-domain for 30 protrusions along the I2 axes and 60 protrusions along the Q2 axes, respectively, on *T* = 3 HEV of the family *Hepeviridae*, similar to *Caliciviridae* [33,34]. We propose that the organization of betanodavirus in the family *Nodaviridae* is intermediate between the families of *Tombusviridae*, *Caliciviridae* and *Birnaviridae* through evolutionary lineage.

### The calcium-incorporating trimeric P-domains

Previous studies have indicated that the surface protrusions on a viral capsid play a crucial role in antigenicity and endocytosis as a result of receptor interactions during virus infection [22,34]. Our high-resolution structure of the truncated P-domain provides not only a structural framework to investigate the particle formation, but also the aetiological basis of host fish-species specificity. We show that the individual P-domain contains a significant _273_ **D**x**D**_275_ motif for calcium binding (Fig 3A and 3C). One Zn^2+^ ion involved in the trimeric organization of VP6 on the rotavirus was previously found at the bottom of the protrusion and near the S-domain along the Q3 axes [42]. In contrast to the rotavirus, this asymmetrical arrangement of two Ca^2+^ ions and two water molecules coordinating with three sets of the _273_ **D**x**D**_275_ motif in the truncated P-domain structure might exist on 60 protrusions of the native *T* = 3 GNNV. We demonstrate that Ca^2+^ plays a significant role in the trimerization of P-domains (S6B Fig). Two metal-binding regions – _130_ **D**xx**D**xD_135_ of the S-domain and _273_ **D**x**D**_275_ of the P-domain–might be essential for the organization and stabilization of *T* = 3 GNNV. In addition to Ca^2+^, conserved water molecules are consistent and integral components of the interfaces between neighboring subunits. These water molecules constitute the primary components of the GNNV protrusion for stabilization through a network of hydrogen bonds (Fig 3C and S6A Fig). In addition, a structural comparison reveals that the truncated P-domain contains the rigid P-domain with the disordered linker region, and this linker region of *T* = 3 GNNV-LP also exhibits large *B*-factor values (S7C Fig). This analysis may provide insights into why the flexible linker region allows the entire solid P-domain to be malleable, resulting in the broken electron density of the P-domain with large *B*-factor values for the *T* = 3 GNNV-LP.

### Functional P-domain for trimerization of CPs, host-cell binding and specificity

Oligomerization of CPs is the first intermediate step in capsid assembly. Based on SEC analysis, the trimeric truncated P-domains appear in the presence of Ca^2+^ (S6B Fig). Interestingly, we observe trimerization of the full-length GNNV CP (112 kDa) and the N-ARM deletion mutant (100 kDa) as well as dimerization of the delta-P-domain mutant (40 kDa) using SDS-PAGE (S6C Fig). Compared with the full-length GNNV CP and the N-ARM deletion mutant, only the *T* = 1 delta-P-domain mutant might exhibit the dimeric capsomer formation in the assembly process, which is similar to that in some RNA plant viruses as well as the Orsay virus, which exhibits the trimeric protrusion in solution (Fig 5B) [19,20,24]. These results suggest that the P-domain may play a major role in promoting trimerization of the GNNV CPs in the initial assembly processes of the *T* = 3 GNNV and the *T* = 1 N-ARM deletion mutant under a Ca^2+^ environment (Fig 5B and S6B and S6C Fig).

The genus betanodavirus is generally classified into four genotypes: SJNNV, BFNNV, TPNNV and RGNNV. A comparison of the genetic heterogeneity of each genotype indicates that the P-domain is a major distinct region [10]. Several hypervariable regions on the P-domain coincide with the protrusion surface associated with the functionalities of the receptor binding and host-cell specificity (Fig 3D) [48]. This observation suggests an evolutionary divergence, resulting in distinct phenotypes of betanodavirus with various fish-host specificities.

Heparan sulphate proteoglycans (HSPs) are negatively charged components of the cell surface and play a role in virion attachment to host cells and binding to secondary host receptors during viral infection. For instance, the surface L-protein of HBV is reported to bind to glycosaminoglycans (GAGs) on the host-cell surface; its GAG-dependent binding is enhanced by PEG to facilitate viral infection [36]. Our study identifies a PEG-binding site on the P-domain of GNNV CP and confirms the enhancement of GNNV infection in presence of PEG (Fig 3E). The heparin-binding ability of GNNV CP has also been demonstrated using immobilized heparin-affinity chromatography [49]. Interestingly, our qPCR analysis of virus copies in GF-1 cells with heparin-containing medium detected no significant signal, suggesting that the presence of heparin suppressed GNNV infection as well. Taken together, GNNV infection might be similar to HBV infection in that they both require an initial attachment to the carbohydrate side-chains of HSPs. Furthermore, the hydrophobic moiety of PEG incorporated on the GNNV P-domain might improve the penetration of non-enveloped viruses across the cell membrane [50].

### N-ARM and particle polymorphism in GNNV assembly

The organization of the N-terminus and encapsidated RNA have been implicated in providing a dynamic equilibrium of the dimeric subunits between “bent” and “flat” conformations during viral assembly [26]. The N-terminus of GNNV CP is composed of the disordered N-ARM and the ordered N-arm comprising the β-annulus, similar to SeMV [20,21,38], and plays a role in regulating *T* = 3 capsid assembly. However, the bent conformation of the subunit dimer leads to the disordered N-arm lying at the inner cavity of the *T* = 1 delta-P-domain mutant without β-annulus formation. This observation indicates that the N-ARM of GNNV CP makes an essential contribution to the organization of the β-annulus along the I3 axes. We propose that the β-annulus on *T* = 3 GNNV might be an outcome of *T* = 3 capsid assembly rather than a profound effect on switching structural symmetries.

Both crystal structures of the N-ARM deletion mutant and the delta-P-domain mutant without the N-ARM of GNNV reveal exclusively *T* = 1 architecture. Comparatively, in alphanodavirus, a complete N-ARM deletion (delta residues 1−54) leads to the inhibition of particle assembly. Conversely, the partial N-ARM deletion (delta residues 1−31) was shown to cause the formation of highly heterogeneous particles, including small bacilliform-like and irregular structures [17]. Furthermore, particle polymorphism of cowpea chlorotic mottle virus (CCMV) was previously described [51], and its N-ARM (residues 1−25) was invisible in the crystal structure [52]. A N-terminal domain deletion mutant (delta residues 1−34) of the CCMV CP resulted in three categories of particles: *T* = 3 VLPs and two SVPs of *T* = 2 and *T* = 1 architectures *in vitro* [53]. The disordered N-ARM might be a critical structural feature of a molecular switch for controlling particle assembly, but this phenomenon was not found in the case of the Orsay virus [24]. A sequence comparison of CPs in the family *Nodaviridae* shows that the Orsay virus CP contains a basic-charged N-terminus but lacks the N-ARM (S8C Fig). The N-terminal deletion mutant of Orsay virus forms a *T* = 3 architecture similar to full-length CP. It is therefore reasonable to assume that the cumulative number of Arg residues on CP might form a proper N-ARM and lead to the spontaneous self-assembly of particle polymorphism, or even the failure of particle assembly.

The *T* = 1 delta-P-domain mutant without Ca^2+^ incorporated shows that the region containing the residues that potentially coordinate Ca^2+^ exhibits an expanded geometry, decreasing subunit contacts along the two-, three- and five-fold axes, and a flatter dimeric contact of curvature ~155°, compared with *T* = 3 GNNV-LP (Fig 4B and 4C). A mutational analysis of Asp residues on SeMV has previously showed that Ca^2+^ coordination is unnecessary for capsid assembly but essential for capsid stability [31]. This is consistent with the observations that Ca^2+^ ions participate in the stability of the GNNV capsid but are not critical for the formation of *T* = 3 or *T* = 1 particles. Taken together, the N-ARM precedes other primary structural components, such as the β-annulus, P-domain and Ca^2+^, to be a molecular switch to ensure the error-free *T* = 3 GNNV assembly.

In summary, this work provides several important structural insights into the genus betanodavirus GNNV. Despite conservation of a viral genome encoding three major proteins and a compatible geometry of the *T* = 3 architecture in the family *Nodaviridae*, the structure of the GNNV-LP obtained here allows us to delineate the key structural components that trigger the oligomerization and stabilize the capsid assembly. Although the jelly-roll fold of the S-domain and the structure of the β-annulus of GNNV capsid are similar to those of known *T* = 3 RNA plant viruses, GNNV exhibits different fold-classifications of the N-arm and the calcium-incorporating trimeric P-domains with a specific DxD motif for trimerization of CPs. The GNNV structure also shows that the hypervariable surface regions of the P-domain contribute to host binding and specificity. The molecular organizations and assembly mechanisms of GNNV reveal that the genus betanodavirus in the family *Nodaviridae* may belong to a significant genus under the viral evolutional pathway among the *Tombusviridae*, *Caliciviridae* and *Birnaviridae* families. Structural mapping of the GNNV P-domain might be useful for the development of vaccine strategies in the fish aquaculture industry.

## Methods

### Ethics statement

All animal experiments were performed in strict accordance with the recommendations in the guide for the Institutional Animal Care and Use Committee, National Cheng Kung University. The protocol was approved under the Institutional Animal Care and Use Committee (IACUC) of National Cheng Kung University (IACUC #100065).

### Production and purification of GNNV particle and truncated GNNV CPs

A consensus CP DNA sequence from the orange-spotted grouper nervous necrosis virus (OSGNNV) RNA2 (GenBank accession no KT071606) was amplified by PCR and cloned into a modified pET32-Xa/LIC vector carrying 6×histidine residues and yeast SUMO (SMT3) as the N-terminal fusion tag [54]. This construct was expressed in *E*. *coli* BL21-CodonPlus(*DE3*)-RIL (Stratagene), and the cells were cultured in Luria Bertani (LB) broth (Merck) containing chloramphenicol (34 μg/ml) and ampicillin (100 μg/ml) until the OD reached 0.6–0.7 at 600 nm at 37°C. IPTG (isopropyl β-D-thiogalactopyranoside) (Bioshop) was added to a final concentration of 0.5 mM and cultures were incubated overnight at 18°C. The cells were harvested and disrupted by sonication in lysis buffer (50 mM Tris HCl (pH 8.0), 0.25 M NaCl, 20 mM imidazole, 5 mM β-mercaptoethanol and 1 mM EGTA). CP was purified through a Ni-NTA column (GE Healthcare). The SUMO-tag was cleaved using SUMO protease that was later removed with a Ni-NTA column.

The purified GNNV CP was diluted to a concentration of 0.3 mg/ml and dialyzed overnight at 4°C against lysis buffer without EGTA or β-mercaptoethanol at a ratio of 1:150. (NH_4_)_2_SO_4_ (750 mM) was added to the dialysis, and GNNV CP was finally dialyzed against the GNNV-LP formation buffer (20 mM Tris HCl (pH 8.0), 0.2 M NaCl, 1% (v/v) glycerol and 2 mM CaCl_2_). The size of GNNV-LP was measured by size-exclusion chromatography on a Superose 6 10/300 GL column (GE Healthcare). The purified GNNV-LP was concentrated to 30 mg/ml and stored at 4°C.

The truncated P-domain (residues 214−338), delta-P-domain mutant (residues 35−217) and N-ARM deletion mutant (residues 35−338) proteins were prepared using the same methods described above for GNNV CP. The truncated P-domain (20 mg/ml) and S-domain (30 mg/ml) proteins were stored in a buffer containing 300 mM NaCl and 50 mM Tris HCl (pH 7.5), whereas the N-ARM deletion mutant (30 mg/ml) was stored in the GNNV-LP formation buffer at 4°C.

### Electron-microscopy analysis of GNNV particles

The purified GNNV-LP, the N-ARM deletion mutant and the delta-P-domain mutant were all diluted to a final concentration of 50 μg/ml and blotted on freshly glow-discharged, carbon-coated 200 mesh copper grids (NISSHIN EM Co, Ltd., Tokyo, Japan). Grids were negatively stained with 5 μl of 2% (w/v) uranyl acetate solution and screened using the H-7650 transmission electron microscope (Hitachi High-Technologies Co.) operated at 80 kV. All images were acquired using a 1024 x 1024 pixels CCD camera (TVIPS, Gauting, Germany) and recorded at a magnification of 100,000 ×.

### Crystallization and X-ray data collection

The initial GNNV-LP crystallization experiment was performed at 18°C with the hanging-drop vapor-diffusion method. A Mosquito liquid-handling robot (TTP Labtech) was used for high-throughput crystallization condition screening. The initial condition of 0.2 M sodium formate (pH 7.2) and 20% (w/v) PEG3350 was obtained from the PEG/Ion Screen I kit (Hampton Research). This condition was further optimized to improve the diffraction quality and resolution of the crystals. Crystals appeared within 1−2 weeks. All crystals were cryoprotected with 25~30% (w/v) PEG3350 and frozen in liquid nitrogen before data collection. X-ray diffraction data were collected on BL44XU at SPring-8 (Harima, Japan) with a CCD detector (MX225-HE, Rayonix) using X-ray wavelength of 0.9 Å. All images were collected with an oscillation angle of 0.3° per frame with an exposure time of 3 s and a crystal-to-detector distance of 600 mm. A total of 600 frames were recorded on different positions from one crystal (0.3 x 0.1 x 0.1 mm^3^). All diffraction data were processed with *HKL2000* [55]. The GNNV-LP crystals belong to a monoclinic *C*2 space group with unit-cell dimensions of *a* = 477 Å, *b* = 422 Å, *c* = 337 Å, and β = 134°. The diffraction data of GNNV-LP crystals contained 499,184 reflections and was 98% complete at a resolution range from 50 to 3.6 Å. To help initial phase determination by *ab initio* phasing [56], the very low-resolution data of the GNNV-LP crystals up to 266 Å were measured, and only a few reflections were not measured in the region of very low resolution (> 100 Å).

The initial crystallizations of the truncated P-domain, delta-P-domain mutant and N-ARM deletion mutant proteins were performed with similar approaches as for the GNNV-LP. The initial crystallization conditions of the truncated P-domain, the delta-P-domain mutant and the N-ARM deletion mutant were 0.2 M Ca acetate, 0.1 M MES (pH 6.5), 10% (w/v) PEG8000; 0.2 M MgCl_2_, 0.1 M HEPES−Na (pH 7.5), 30% (w/v) PEG400; and 0.1 M NaCl, 0.1M lithium sulfate, 0.1 M MES (pH 6.5), 30% (w/v) PEG400, respectively. All crystals appeared within one week. X-ray diffraction data of the truncated P-domain and delta-P-domain mutants were collected on BL15A1 with a CCD detector (MX300-HE, Rayonix) of NSRRC in Taiwan at a wavelength of 1.0 Å. The N-ARM deletion mutant was collected on BL44XU at SPring-8 (Harima, Japan) with a CCD detector (MX300-HE, Rayonix) at a wavelength of 0.9 Å. The diffraction data were processed with *HKL2000* [55]. All data processing statistics are shown in Table 1.

### Crystal structure determination and refinement

The initial phases of the *T* = 3 GNNV-LP were determined by the *ab initio* method using icosahedral non-crystallographic symmetry (NCS) averaging [56]. Self-rotation functions of *κ* = 72°, 120° and 180° hemispheres were analyzed with *Molrep* [57] to confirm the icosahedral symmetries of GNNV-LP crystals and to determine the orientation of the icosahedral symmetry. There were two *T* = 3 GNNV-LP particles in the monoclinic unit cell with one two-fold NCS axis of the virus particle coinciding with the crystallographic two-fold axis. The asymmetric unit contained half of the particle or 30 copies of the icosahedrally-related trimeric CPs. The spherical-shell model with uniform density was used as the starting model. The inner and outer radii of 119 and 159 Å, respectively, were chosen as initial parameters of the model [56]. For the *ab initio* method, which used NCS-averaging (NCSA) with phase extension, a proper mask was necessary for dividing two regions: the protein region to be NCS-averaged and the solvent region to be flattened. The initial mask for NCSA and solvent flattening was created from the atomic structure of *T* = 3 FHV (PDB ID: 4FSJ) with a large mask-radius of 11~13 Å around each atoms (S1A Fig). The initial NCS operators for averaging were derived from the self-rotation function. In a basic NCSA cycle between dual spaces, 30-fold NCSA and solvent flattening were applied in real space followed by phase combination with the Rayment weighting [58] in reciprocal space. In most of the procedure, programs from *RAVE* [59] and *CCP4* [60] were used. After more than one hundred cycles of iteration at 25 Å resolution, the phase extension was performed from 25 Å to 3.7 Å with 50 iterations in one reciprocal lattice step (≈ *1/a*) (This process is referred as “procedure” hereafter). During cycles of iterations, the *R* factor and correlation coefficient comparing *F*_obs_ and *F*_calc_ were monitored. The interpretable electron density map was successfully obtained (S1A, S1B and S1C Fig) by this procedure. To improve the electron density, the mask was updated based on the resultant map. The NCS operators were refined from the orientation of the icosahedral symmetry to give the highest correlation coefficient. The procedures were started from the spherical-shell uniform density model with the updated mask and NCS operators. The best values of the *R* factor and the correlation coefficient appeared to be 0.20 and 0.92, respectively, at ~6 Å resolution. The overall values of these calculations are given in Table 1 and the progress of the phase extension is shown in S1D Fig The enantiomorph of the phase set was checked by the electron density of the helical structural elements (S1C Fig).

In the last cycle of phase improvement, *DM* [61] was used for NCSA with refinement of NCS operators, and resolution was extended to 3.1 Å. Although diffraction data higher than 3.6 Å resolution was of poor quality, the electron density map calculated with the phases extended to 3.1 Å resolution gave the result better than the map calculated with the data extended to 3.6 Å. Quality of the final electron density maps was good enough for an atomic model building except the P-domain region of *T* = 3 GNNV-LP. We suspected that the P-domain of *T* = 3 GNNV-LP did not follow the strict icosahedral symmetry. However, utilizing of *DM* with various trials, including the individual mask and NCS operator around the P-domain, did not significantly improve the density map around the P-domain of *T* = 3 GNNV-LP.

The initial model building of GNNV-LP was performed by Cα-tracing with *Coot* [62] from the *DM* maps (3.1 Å). The complete models of the S-domain and the linker region were subsequently built up based on the amino-acid sequence of the GNNV CP manually (S1C Fig).

Structure refinement of the *T* = 3 GNNV-LP was performed using *REFMAC5* [63] with icosahedral NCS restraints. During the refinement, the additional restraint was required for the coordinates of the P-domain to avoid the divergence due to the poor electron density. The *PROSMART* [64] with the high-resolution truncated P-domain model, which was subsequently determined, was used as the initial model and restraint reference. The coordinates were refined to a crystallographic *R*_cryst_ of 0.257 and *R*_free_ of 0.295 at 3.6 Å resolution. Analysis of the Ramachandran plot showed that 97% of the main-chain dihedral angles were in preferred regions; 3% was in the allowed regions; and none were in the outlier regions using *MolProbity* [65]. The results of the GNNV-LP structure determination are summarized in Table 1.

For structure determination of the *T* = 1 delta-P-domain mutant, the coordinate of the S-domain from the *T* = 3 GNNV-LP structure was used as the molecular-replacement initial model. The icosahedral 20-fold NCSA phase extension by *DM* [59] was used for phase improvement. The structural model of the *T* = 1 delta-P-domain mutant was refined with the NCS restraints using *REFMAC5* [63] and manual revision using *Coot* [62] to fit the *DM* map. The electron density of the P-domain, which was cut out from the density map of the *T* = 3 GNNV-LP, was used as the search model for molecular replacement of the truncated P-domain crystal. After phase improvement by the multiple crystal averaging with self-made programs together with *MAPROT* [66], the model building was automatically performed with *ARP/wARP* [67]. Structure refinement of the truncated P-domain was performed with *PHENIX* [68]. Resolution of the data for the N-ARM deletion mutant was rather modest at 7 Å. The rough structure of the N-ARM deletion mutant was obtained as a reasonable MR solution using *PHASER* [69]. The crystal packing and self-rotation function analyses are shown in S3A and S3B Fig. In the figures, one MR solution is shown, in which two particles are located at (1/3, 2/3, 1/4) and (2/3, 1/3, 3/4) in one unit cell. All graphics for the molecular structure were produced with the *PyMOL* (http://www.pymol.org/).

### Cell culture and virus infection

The GF-1 grouper cell line [70] was cultured in antibiotic-free Leibovitz’s L-15 medium (Gibco) supplemented with 5% (v/v) fetal bovine serum (FBS) at 28°C. GNNV was isolated from naturally infected groupers (*Epinephelus coioides*) collected in Taiwan. The isolated virus was propagated in GF-1 cells and collected when 90% of the cells displayed a cytopathic effect (CPE). GF-1 cells were seeded in 12-well plates at a density of 1 x 10^5^ per well in 2 ml L-15 medium supplemented with 5% (v/v) FBS, and cultured to 80−90% confluence. For infection, GF-1 cells were washed with PBS three times and subsequently infected with GNNV at a titer of 10^4^ TCID_50_/ml in serum-free medium, 4% (w/v) PEG3000- and 4% (w/v) PEG8000-containing serum-free medium, respectively. After incubation with the virus for 30 min, the cells were washed thrice with PBS and L-15 medium (2 ml) supplemented with 1% (v/v) FBS in the presence or absence of PEG3000 and PEG8000 was added.

### qPCR analysis of GNNV RNA and statistics

After infection, GF-1 cells were washed with PBS, and RNA was extracted with the TRIzol reagent (Invitrogen). Reverse transcription and real-time quantitative PCR were performed as previously described [71]. All data analyses were shown as mean ± SD of three independent experiments. Statistical analyses were assessed by one-way ANOVA with SPSS statistical software version 17.0 (SPSS Inc.). P values < 0.05, were considered statistically significant.

### Accession numbers

Nucleotide sequences of PCR-amplified fragments of OSGNNV RNA2 from have been deposited in the GenBank nucleotide database under the accession code KT071606. Atomic coordinates and diffraction data of the *T* = 3 GNNV-LP, the truncated P-domain (214−338) and the *T* = 1 delta-P-domain mutant (35−217) have been deposited at the Protein Data Bank (PDB) with accession codes 4WIZ, 4RFU and 4RFT, respectively.

## Supporting Information

S1 Fig Structural determination of the *T* = 3 GNNV-LPs by the *ab initio* method with NCSA. (A) Lateral view of the iASU of *T* = 3 GNNV-LP (blue, final refined model) and FHV (green, located at the place making the initial mask) and the first (‘initial’) interpretable electron density map at 3.7 Å and the ‘initial’ mask made from the *T* = 3 FHV (orange). (B) Inner surface view of the density map at 3.7 Å corresponding to the iASU, labeled accordingly with the Cα carbon skeletons of CP of GNNV (blue) and FHV (green) superimposed. RNA-binding α-helices of FHV are removed for clear view. (C) A stereo-view of the α-helix (residues 90–99) in the S-domain directed towards the corresponding ‘initial’ density map at 3.7 Å. (D) Progress of the *R* factors and correlation coefficients during phase extension. The ‘initial’ means the first interpretable trial by the *ab initio* method. The ‘final’ means the progress during the phase extension with the revised mask and the refined NCSA matrices.
(TIFF)

S2 Fig EM images of GNNV-LPs of different capsid organization. Electron micrographs of negatively stained VLPs used for crystallization. (A) *T* = 3 GNNV-LPs; (B) *T* = 1 SVPs of the N-ARM deletion mutant; (C) the delta-P-domain mutant. Bar: 100 nm.
(TIFF)

S3 Fig Crystal packing and self-rotation function of the *T* = 1 N-ARM deletion mutant. (A) Crystal packing of N-ARM deletion mutant in *T* = 1 assembly is well arranged in the corresponding unit cell with dimensions of *a* = *b* = 260.8 Å, *c* = 250.5 Å and γ = 120° in space group *P*6_3_22. Five neighboring subunits of CP along the I5 axis are labeled as green, wheat, cyan, yellow and magenta colors, respectively. (B) Analyses of self-rotation functions of the *T* = 1 N-ARM deletion mutant. The NCS relationship was corroborated by the self-rotation functions of *κ* = 72°, 120° and 180° hemispheres, and calculated with *Molrep* [57].
(TIFF)

S4 Fig Structural organization of the S-domain. (A) S-domains of three subunits per iASU. Three Ca^2+^ ions (yellow spheres) are incorporated at the interfaces of neighboring subunits A (cyan), B (green) and C (magenta), where Gln100, Asp130, Asp133, Ser170 and Glu213 (orange sticks) participate in Ca^2+^ coordination. (B) An illustration of the geometric and electrostatic differences on the inner surface between GNNV (*left*) and PaV (*right*). The charge distribution around the inner surface of the pentameric capsomers of empty GNNV-LP and PaV has a positive electrostatic potential (blue), and five short ordered encapsidated RNA duplexes are shown along the I2 axes. (C) The distribution of cysteine residues in the S-domains. A superimposition of S-domain structures from GNNV (magenta), SeMV (green, PDB ID: 1X33) and Orsay virus (blue, PDB ID: 4NWV) is shown. Cysteine residues are shown in stick, and Cys107−Cys195 of Orsay virus represents the disulfide linkage.
(TIFF)

S5 Fig Structural comparison of the P-domains among GNNV, Orsay virus, HEV and Calicivirus. The crystal structures of the P-domain of the GNNV and Orsay viruses (PDB ID: 4NWV), and P1-domain of HEV (PDB ID: 2ZTN) and Calicivirus (PDB ID: 2GH8) are shown as ribbon diagrams. The N’ and C’ terminus of Calicivirus indicate the connecting regions between the P1 and P2 domains.
(TIFF)

S6 Fig Specific arrangement of water molecules in the P-domain and the Ca^2+^-mediated oligomerization of GNNV CP. (A) Conservation of water molecules at the interfaces of neighboring subunits in the truncated P-domain. Three sets of two water molecules (blue spheres), at the subunit-​interface regions, are coordinated with the conserved residues shown from two neighboring subunits in yellow and green, respectively. (B) The equilibrium properties of the monomeric and trimeric P-domains. The purified GNNV P-domain was analyzed in the absence (solid line) or presence (dashed line) of Ca^2+^ using size-exclusion chromatography (SEC) on a Superdex 75 10/300 GL column (GE Healthcare). These data were compared to protein standards (conalbumin, 75 kDa; ovalbumin, 43 kDa; ribonuclease A, 13.7 kDa). (C) SDS-PAGE analysis of oligomerization of three CPs in solution. Full-length GNNV CP (lane 1) and the N-ARM deletion mutant (residues 35−338) (lane 2) exhibit monomeric and trimeric forms concurrently; and monomeric and dimeric forms of the delta-P-domain mutant (residues 35−217) (lane 3) are shown compared to protein standards (lane M).
(TIFF)

S7 Fig The Ca^2+^-binding and ligand-binding pockets of the P-domain. (A) A stereo view of the trimeric P-domains with Ca^2+^ ions and water molecules bound at the **D**x**D** motif. Two residues (Asp273 and Asp275, in sticks) coordinating two Ca^2+^ ions (yellow spheres) and water molecules (blue spheres) are conserved on the P-domain of each neighboring subunit. The |2*F*_o_–*F*_c_| map (orange mesh) around the binding site is shown with a contour level at 3σ. (B) There are three conserved GOL-binding pockets and one PEG-binding site on the electrostatic surface of the trimeric P-domains. Electron density maps of PEG (blue) and GOL (green) are shown with the |2*F*_o_–*F*_c_| coefficient and contour at 1σ around the interaction site. (C) A comparison of the linker regions between the truncated P-domain and the *T* = 3 GNNV-LP. Each hinge region is identified and color-coded as a rainbow gradient with respect to *B*-factor values (blue, lowest; red, highest) to depict the relatively rigid and flexible area. The three subunits (A, B and C) are shown with magenta, green and cyan, respectively.
(TIFF)

S8 Fig Phylogenetic tree and sequence alignment of CPs of the family *Nodaviridae*. (A) The phylogenetic tree of the family *Nodaviridae*. The neighbor-joining phylogenetic unrooted tree was built using Mega5 [72] with a multiple alignment of conserved blocks of the sequence of RNA2. The major clades of the family *Nodaviridae* are identified as alphanodavirus, betanodavirus, unassigned nodavirus and Orsay virus, respectively. (B) The sequence alignment of RNA2-encoded CP from different genotypes of betanodavirus. Multiple sequence alignment was performed with sequences of the CPs from OSGNNV, DGNNV, RGNNV, BFNNV, SJNNV and TPNNV using ClustalW. Each domain of GNNV CP is indicated on the top of alignment with colors as in Fig 1B. The **D**xx**D**xD and **D**x**D** motifs of GNNV CP are identified in the orange boxes. (C) N-terminal sequence identification of the CPs from different strains of the family *Nodaviridae*. Basic- and acidic-charged residues are colored in blue and red, respectively.
(TIFF)

## References

[1] Ahlquist P. RNA-dependent RNA polymerases, viruses, and RNA silencing. Science. 2002;296: 1270–1273. 12016304 10.1126/science.1069132

[2] Ahlquist P. Parallels among positive-strand RNA viruses, reverse-transcribing viruses and double-stranded RNA viruses. Nat Rev Microbiol. 2005;4: 371–382. 10.1038/nrmicro1389 PMC7097367 16582931

[3] Chao JA, Lee JH, Chapados BR, Debler EW, Schneemann A, Williamson JR. Dual modes of RNA-silencing suppression by Flock house virus protein B2. Nat Struct Mol Biol. 2006;12: 952–957. 10.1038/nsmb1005 16228003

[4] Li Y, Lu JF, Han YH, Fan XX, Ding SW. RNA interference functions as an antiviral immunity mechanism in mammals. Science. 2013;342: 231–234. 10.1126/science.1241911 24115437 PMC3875315

[5] Lu R, Maduro M, Li F, Li HW, Broitman-Maduro G, Li WX, et al Animal virus replication and RNAi-mediated antiviral silencing in *Caenorhabditis elegans* . Nature. 2005;436: 1040–1043. 16107851 10.1038/nature03870 PMC1388260

[6] Ou MC, Chen YM, Jeng MF, Chu CJ, Yang HL, Chen TY. Identification of critical residues in nervous necrosis virus B2 for dsRNA-binding and RNAi-inhibiting activity through by bioinformatic analysis and mutagenesis. Biochem Bioph Res Co. 2007;361: 634–640. 10.1016/j.bbrc.2007.07.075 17669362

[7] Thiery R, Cozien J, de Boisseson C, Kerbart-Boscher S, Nevarez L. Genomic classification of new betanodavirus isolates by phylogenetic analysis of the coat protein gene suggests a low host-fish species specificity. J Gen Virol. 2004;85: 3079–3087. 15448371 10.1099/vir.0.80264-0

[8] Yoshikoshi K, Inoue K. Viral nervous necrosis in hatchery-reared larvae and juveniles of Japanese parrotfish, *Oplegnathus fasciatus* (Temminck and Schlegel). J Fish Dis. 1990;13: 69–77.

[9] Munday BL, Kwang J, Moody N. Betanodavirus infections of teleost fish: a review. J Fish Dis. 2002;25: 127–142.

[10] Nishizawa T, Furuhashi M, Nagai T, Nakai T, Muroga K. Genomic classification of fish nodaviruses by molecular phylogenetic analysis of the coat protein gene. Appl Environ Microb. 1997;63: 1633–1636. 10.1128/aem.63.4.1633-1636.1997 PMC168456 9097459

[11] Schuster S, Zirkel F, Kurth A, van Cleef KWR, Drosten C, van Rij RP, et al A unique nodavirus with novel features: mosinovirus expresses two subgenomic RNAs, a capsid gene of unknown origin, and a suppressor of the antiviral RNA interference pathway. J Virol. 2014;88: 13447–13459. 10.1128/JVI.02144-14 25210176 PMC4249075

[12] Tang KF, Pantoja CR, Redman RM, Navarro SA, Lightner DV. Ultrastructural and sequence characterization of *Penaeus vannamei* nodavirus (PvNV) from Belize. Dis Aquat Organ. 2011;94: 179–187 10.3354/dao02335 21790065

[13] Cheng SS, Brooks CL. Viral capsid proteins are segregated in structural fold space. PLoS Comput Biol. 2013;9: e1002905 10.1371/journal.pcbi.1002905 23408879 PMC3567143

[14] Schneemann A, Zhong WD, Gallagher TM, Rueckert RR. Maturation cleavage required for infectivity of a nodavirus. J Virol. 1992;66: 6728–6734. 1404613 10.1128/jvi.66.11.6728-6734.1992 PMC240169

[15] Tang L, Johnson KN, Ball LA, Lin TW, Yeager M, Johnson JE. The structure of Pariacoto virus reveals a dodecahedral cage of duplex RNA. Nat Struct Biol. 2001;8: 77–83. 11135676 10.1038/83089

[16] Fisher AJ, Johnson JE. Ordered duplex RNA controls capsid architecture in an icosahedral animal virus. Nature. 1993;361: 176–179. 8421524 10.1038/361176a0

[17] Dong XF, Natarajan P, Tihova M, Johnson JE, Schneemann A. Particle polymorphism caused by deletion of a peptide molecular switch in a quasi-equivalent icosahedral virus. J Virol. 1998;72: 6024–6033. 9621065 10.1128/jvi.72.7.6024-6033.1998 PMC110407

[18] Tang L, Lin CS, Krishna NK, Yeager M, Schneemann A, Johnson JE. Virus-like particles of a fish nodavirus display a capsid subunit domain organization different from that of insect nodaviruses. J Virol. 2002;76: 6370–6375. 12021370 10.1128/JVI.76.12.6370-6375.2002 PMC136213

[19] Qu CX, Liljas L, Opalka N, Brugidou C, Yeager M, Beachy RN, et al 3D domain swapping modulates the stability of members of an icosahedral virus group. Structure. 2000;8: 1095–1103. 11080631 10.1016/s0969-2126(00)00508-6

[20] Pappachan A, Subashchandrabose C, Satheshkumar PS, Savithri HS, Murthy MRN. Structure of recombinant capsids formed by the beta-annulus deletion mutant–rCP (Delta 48–59) of Sesbania mosaic virus. Virology. 2008;375: 190–196. 10.1016/j.virol.2008.01.023 18295296

[21] Satheshkumar PS, Lokesh GL, Murthy MRN, Savithri HS. The role of arginine-rich motif and beta-annulus in the assembly and stability of Sesbania mosaic virus capsids. J Mol Biol. 2005;353: 447–458. 16169007 10.1016/j.jmb.2005.08.021

[22] Chen R, Neill JD, Estes MK, Prasad BVV. X-ray structure of a native calicivirus: structural insights into antigenic diversity and host specificity. Proc Natl Acad Sci USA. 2006;103: 8048–8053. 16702551 10.1073/pnas.0600421103 PMC1472427

[23] Holm L, Sander C. Dali: a network tool for protein-structure comparison. Trends Biochem Sci. 1995;20: 478–480. 8578593 10.1016/s0968-0004(00)89105-7

[24] Guo YR, Hryc CF, Jakana J, Jiang H, Wang D, Chiu W, et al Crystal structure of a nematode-infecting virus. Proc Natl Acad Sci USA. 2014;111: 12781–12786. 10.1073/pnas.1407122111 25136116 PMC4156749

[25] Morgunova EY, Dauter Z, Fry E, Stuart DI, Stelmashchuk VY, Mikhailov AM, et al The atomic-structure of Carnation Mottle Virus capsid protein. Febs Lett. 1994;338: 267–271. 8307192 10.1016/0014-5793(94)80281-5

[26] Prasad BVV, Schmid MF. Principles of virus structural organization. Adv Exp Med Biol. 2012;726: 17–47. 10.1007/978-1-4614-0980-9_3 22297509 PMC3767311

[27] Banerjee M, Speir JA, Kwan MH, Huang R, Aryanpur PP, Bothner B, et al Structure and function of a genetically engineered mimic of a nonenveloped virus entry intermediate. J Virol. 2010;84: 4737–4746. 10.1128/JVI.02670-09 20164221 PMC2863772

[28] Li M, Kakani K, Katpally U, Johnson S, Rochon D, Smith TJ. Atomic structure of Cucumber necrosis virus and the role of the capsid in vector transmission. J Virol. 2013;87: 12166–12175. 10.1128/JVI.01965-13 24006433 PMC3807921

[29] Harrison SC, Olson AJ, Schutt CE, Winkler FK, Bricogne G. Tomato bushy stunt virus at 2.9-a resolution. Nature. 1978;276: 368–373. 19711552 10.1038/276368a0

[30] Hogle J, Kirchhausen T, Harrison SC. Divalent-cation sites in tomato bushy stunt virus—difference maps at 2.9 Å resolution. J Mol Biol. 1983;171: 95–100. 6417343 10.1016/s0022-2836(83)80315-5

[31] Satheshkumar PS, Lokesh GL, Sangita V, Saravanan V, Vijay CS, Murthy MRN, et al Role of metal ion-mediated interactions in the assembly and stability of Sesbania mosaic virus T = 3 and T = 1 capsids. J Mol Biol. 2004;342: 1001–1014. 15342252 10.1016/j.jmb.2004.07.022

[32] Wery JP, Reddy VS, Hosur MV, Johnson JE. The refined three-dimensional structure of an insect virus at 2.8 Å resolution. J Mol Biol. 1994;235: 565–586. 8289282 10.1006/jmbi.1994.1014

[33] Guu TSY, Liu Z, Ye QZ, Mata DA, Li KP, Yin CC, et al Structure of the hepatitis E virus-like particle suggests mechanisms for virus assembly and receptor binding. Proc Natl Acad Sci USA. 2009;106: 12992–12997. 10.1073/pnas.0904848106 19622744 PMC2722310

[34] Yamashita T, Mori Y, Miyazaki N, Cheng RH, Yoshimura M, Unno H, et al Biological and immunological characteristics of hepatitis E virus-like particles based on the crystal structure. Proc Natl Acad Sci USA. 2009;106: 12986–12991. 10.1073/pnas.0903699106 19620712 PMC2722322

[35] Sangita V, Satheshkumar PS, Savithri HS, Murthy MRN. Structure of a mutant T = 1 capsid of Sesbania mosaic virus: role of water molecules in capsid architecture and integrity. Acta Crystallogr D Biol Crystallogr. 2005;61: 1406–1412. 16204894 10.1107/S0907444905024030

[36] Schulze A, Gripon P, Urban S. Hepatitis B virus infection initiates with a large surface protein-dependent binding to heparan sulfate proteoglycans. Hepatology. 2007;46: 1759–1768. 18046710 10.1002/hep.21896

[37] Schneemann A. The structural and functional role of RNA in icosahedral virus assembly. Annu Rev Microbiol. 2006;60: 51–67. 16704342 10.1146/annurev.micro.60.080805.142304

[38] Sangita V, Lokesh GL, Satheshkumar PS, Vijay CS, Saravanan V, Savithri HS, et al T = 1 capsid structures of Sesbania mosaic virus coat protein mutants: determinants of T = 3 and T = 1 capsid assembly. J Mol Biol. 2004;342: 1402–1405. 10.1016/j.jmb.2004.07.003 15342251

[39] Abadzapatero C, Abdelmeguid SS, Johnson JE, Leslie AGW, Rayment I, Rossmann MG, et al Structure of southern bean mosaic virus at 2.8 Å resolution. Nature. 1980;286: 33–39. 19711553 10.1038/286033a0

[40] Silva AM, Rossmann MG. Refined structure of southern bean mosaic-virus at 2.9 Å resolution. J Mol Biol. 1987;197: 69–87. 3681993 10.1016/0022-2836(87)90610-3

[41] Neu U, Khan ZM, Schuch B, Palma AS, Liu Y, Pawlita M, et al Structures of B-lymphotropic polyomavirus VP1 in complex with oligosaccharide ligands. PLoS Pathog. 2013;9: e1003714 10.1371/journal.ppat.1003714 24204265 PMC3814675

[42] Mathieu M, Petitpas I, Navaza J, Lepault J, Kohli E, Pothier P, et al Atomic structure of the major capsid protein of rotavirus: implications for the architecture of the virion. EMBO J. 2001;20: 1485–1497. 11285213 10.1093/emboj/20.7.1485 PMC145492

[43] Wu YM, Hsu CH, Wang CH, Liu WT, Chang WH, Lin CS. Role of the DxxDxD motif in the assembly and stability of betanodavirus particles. Arch Virol. 2008;53: 1633–1642. 10.1007/s00705-008-0150-6 18626568

[44] Chelvanayagam G, Heringa J, Argos P. Anatomy and evolution of proteins displaying the viral capsid jellyroll topology. J Mol Biol 1992;228: 220–242. 1447783 10.1016/0022-2836(92)90502-b

[45] Wang CH, Hsu CH, Wu YM, Luo YC, Tu MH, Chang WH, et al Roles of cysteines Cys115 and Cys201 in the assembly and thermostability of grouper betanodavirus particles. Virus Genes. 2010;41: 73–80. 10.1007/s11262-010-0488-1 20446029 PMC2886913

[46] Sangita V, Lokesh GL, Satheshkumar PS, Saravanan V, Vijay CS, Savithri HS, et al Structural studies on recombinant T = 3 capsids of Sesbania mosaic virus coat protein mutants. Acta Crystallogr D Biol Crystallogr. 2005;61: 1402–1405. 16204893 10.1107/S0907444905024029

[47] Coulibaly F, Chevalier C, Gutsche I, Pous J, Navaza J, Bressanelli S, et al The birnavirus crystal structure reveals structural relationships among icosahedral viruses. Cell. 2005;120: 761–772. 15797378 10.1016/j.cell.2005.01.009

[48] Ito Y, Okinaka Y, Mori KI, Sugaya T, Nishioka T, Oka M, et al Variable region of betanodavirus RNA2 is sufficient to determine host specificity. Dis Aquat Organ. 2008;79: 199–205. 10.3354/dao01906 18589996

[49] Choi YR, Kim HJ, Lee JY, Kang HA, Kim HJ. Chromatographically-purified capsid proteins of red-spotted grouper nervous necrosis virus expressed in *Saccharomyces cerevisiae* form virus-like particles. Protein Expres Purif. 2013;89: 162–168. 10.1016/j.pep.2013.03.007 23537792

[50] Tsai B. Penetration of nonenveloped viruses into the cytoplasm. Annu Rev Cell Dev Biol. 2007;23: 23–43. 17456018 10.1146/annurev.cellbio.23.090506.123454

[51] Bancroft JB. The self-assembly of spherical plant viruses. Adv Virus Res. 1970;16: 99–134. 4924992 10.1016/s0065-3527(08)60022-6

[52] Speir JA, Munshi S, Wang GJ, Baker TS, Johnson JE. Structures of the native and swollen forms of cowpea chlorotic mottle virus determined by X-ray crystallography and cryo-electron microscopy. Structure. 1995;3: 63–78. 7743132 10.1016/s0969-2126(01)00135-6 PMC4191737

[53] Tang JH, Johnson JM, Dryden KA, Young MJ, Zlotnick A, Johnson JE. The role of subunit hinges and molecular "switches" in the control of viral capsid polymorphism. J Struct Biol. 2006;154: 59–67. 16495083 10.1016/j.jsb.2005.10.013

[54] Lee CD, Sun HC, Hu SM, Chiu CF, Homhuan A, Liang SM, et al An improved SUMO fusion protein system for effective production of native proteins. Protein Sci. 2008;17: 1241–1248. 10.1110/ps.035188.108 18467498 PMC2442006

[55] Otwinowski Z, Minor W. Processing of X-ray diffraction data collected in oscillation mode. Method Enzymol. 1997;276: 307–326. 10.1016/S0076-6879(97)76066-X 27754618

[56] Taka J, Naitow H, Yoshimura M, Miyazaki N, Nakagawa A, Tsukihara T. Ab initio crystal structure determination of spherical viruses that exhibit a centrosymmetric location in the unit cell. Acta Crystallogr D Biol Crystallogr. 2005;61: 1099–1106. 16041075 10.1107/S0907444905015866

[57] Vagin A, Teplyakov A. Molecular replacement with MOLREP. Acta Crystallogr D Biol Crystallogr. 2010;66: 22–25. 10.1107/S0907444909042589 20057045

[58] Rayment I. Molecular replacement method at low resolution: optimum strategy and intrinsic limitations as determined by calculations on icosahedral virus models. Acta Crystallogr A. 1983;39: 102–116.

[59] Kleywegt GJ, Jones TA. Software for handling macromolecular envelopes. Acta Crystallogr D Biol Crystallogr. 1999;55: 941–944. 10089342 10.1107/s0907444999001031

[60] Winn MD, Ballard CC, Cowtan KD, Dodson EJ, Emsley P, Evans PR, et al Overview of the CCP4 suite and current developments. Acta Crystallogr D Biol Crystallogr. 2011;67: 235–242. 10.1107/S0907444910045749 21460441 PMC3069738

[61] Cowtan K. Dm: An automated procedure for phase improvement by density modification. Joint CCP4 and ESF-EACBM Newsletter on Protein Crystallography. 1994;31: 34–38.

[62] Emsley P, Lohkamp B, Scott WG, Cowtan K. Features and development of Coot. Acta Crystallogr D Biol Crystallogr. 2010;66: 486–501. 10.1107/S0907444910007493 20383002 PMC2852313

[63] Murshudov GN, Vagin AA, Dodson EJ. Refinement of macromolecular structures by the maximum-likelihood method. Acta Crystallogr D Biol Crystallogr. 1997;53: 240–255. 15299926 10.1107/S0907444996012255

[64] Nicholls RA, Long F, Murshudov GN. Low-resolution refinement tools in REFMAC5. Acta Crystallogr D Biol Crystallogr. 2012;68: 404–417. 10.1107/S090744491105606X 22505260 PMC3322599

[65] Chen VB, Arendall WB, Headd JJ, Keedy DA, Immormino RM, Kapral GJ, et al Molprobity: all-atom structure validation for macromolecular crystallography. Acta Crystallogr D Biol Crystallogr. 2010;66: 12–21. 10.1107/S0907444909042073 20057044 PMC2803126

[66] Stein PE, Boodhoo A, Armstrong GD, Cockle SA, Klein MH, Read RJ. The crystal-structure of pertussis toxin. Structure. 1994;2: 45–57. 8075982 10.1016/s0969-2126(00)00007-1

[67] Langer G, Cohen SX, Lamzin VS, Perrakis A. Automated macromolecular model building for X-ray crystallography using ARP/wARP version 7. Nat Protoc. 2008;3: 1171–1179. 10.1038/nprot.2008.91 18600222 PMC2582149

[68] Adams PD, Afonine PV, Bunkoczi G, Chen VB, Davis IW, Echols N, et al PHENIX: a comprehensive Python-based system for macromolecular structure solution. Acta Crystallogr D Biol Crystallogr. 2010;66: 213–221. 10.1107/S0907444909052925 20124702 PMC2815670

[69] Mccoy AJ, Grosse-Kunstleve RW, Adams PD, Winn MD, Storoni LC, Read RJ. Phaser crystallographic software. J Appl Crystallogr. 2007;40: 658–674. 19461840 10.1107/S0021889807021206 PMC2483472

[70] Chi SC, Hu WW, Lo BJ. Establishment and characterization of a continuous cell line (GF-1) derived from grouper, *Epinephelus coioides* (Hamilton): a cell line susceptible to grouper nervous necrosis virus (GNNV). J Fish Dis. 1999;22: 173–182.

[71] Kuo HC, Wang TY, Chen PP, Chen YM, Chuang HC, Chen TY. Real-time quantitative pcr assay for monitoring of nervous necrosis virus infection in grouper aquaculture. J Clin Microbiol. 2011;49: 1090–1096. 10.1128/JCM.01016-10 21233077 PMC3067732

[72] Tamura K, Peterson D, Peterson N, Stecher G, Nei M, Kumar S. MEGA5: molecular evolutionary genetics analysis using maximum likelihood, evolutionary distance, and maximum parsimony methods. Mol Biol Evol. 2011;28: 2731–2739. 10.1093/molbev/msr121 21546353 PMC3203626

