# Supplementary figures and images for: Crystal Structures of a Piscine Betanodavirus: Mechanisms of Capsid Assembly and Viral Infection
Source: PLoS Pathog. 2015 Oct 22;11(10):e1005203. doi: 10.1371/journal.ppat.1005203 (PMC4619592; doi:10.1371/journal.ppat.1005203)

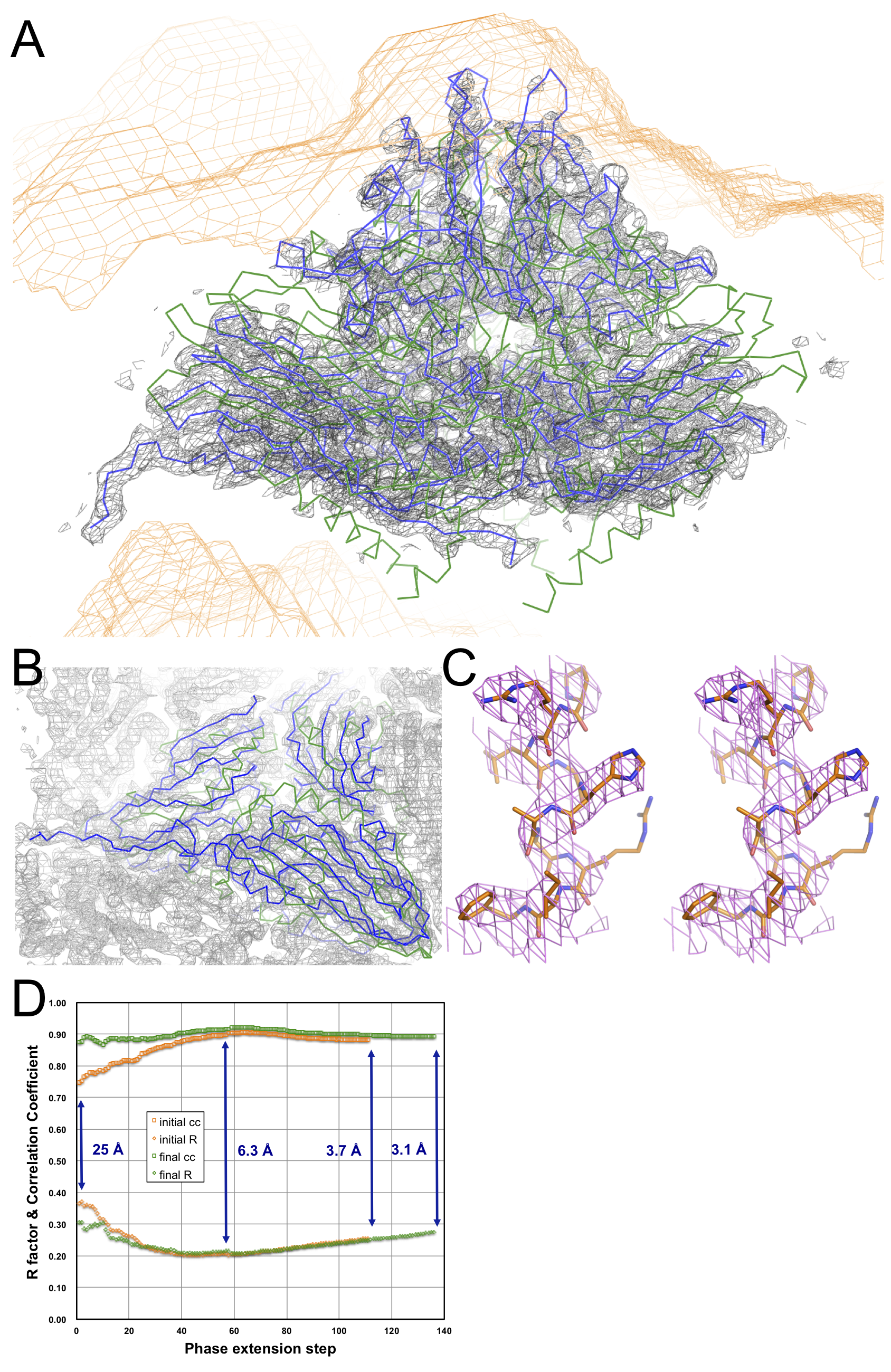

Supplement: S1 Fig — (A) Lateral view of the iASU of T = 3 GNNV-LP (blue, final refined model) and FHV (green, located at the place making the initial mask) and the first (‘initial’) interpretable electron density map at 3.7 Å and the ‘initial’ mask made from the T = 3 FHV (orange). (B) Inner surface view of the density map at 3.7 Å corresponding to the iASU, labeled accordingly with the Cα carbon skeletons of CP of GNNV (blue) and FHV (green) superimposed. RNA-binding α-helices of FHV are removed for clear view. (C) A stereo-view of the α-helix (residues 90–99) in the S-domain directed towards the corresponding ‘initial’ density map at 3.7 Å. (D) Progress of the R factors and correlation coefficients during phase extension. The ‘initial’ means the first interpretable trial by the ab initio method. The ‘final’ means the progress during the phase extension with the revised mask and the refined NCSA matrices. (TIFF) [file ppat.1005203.s001.tiff]

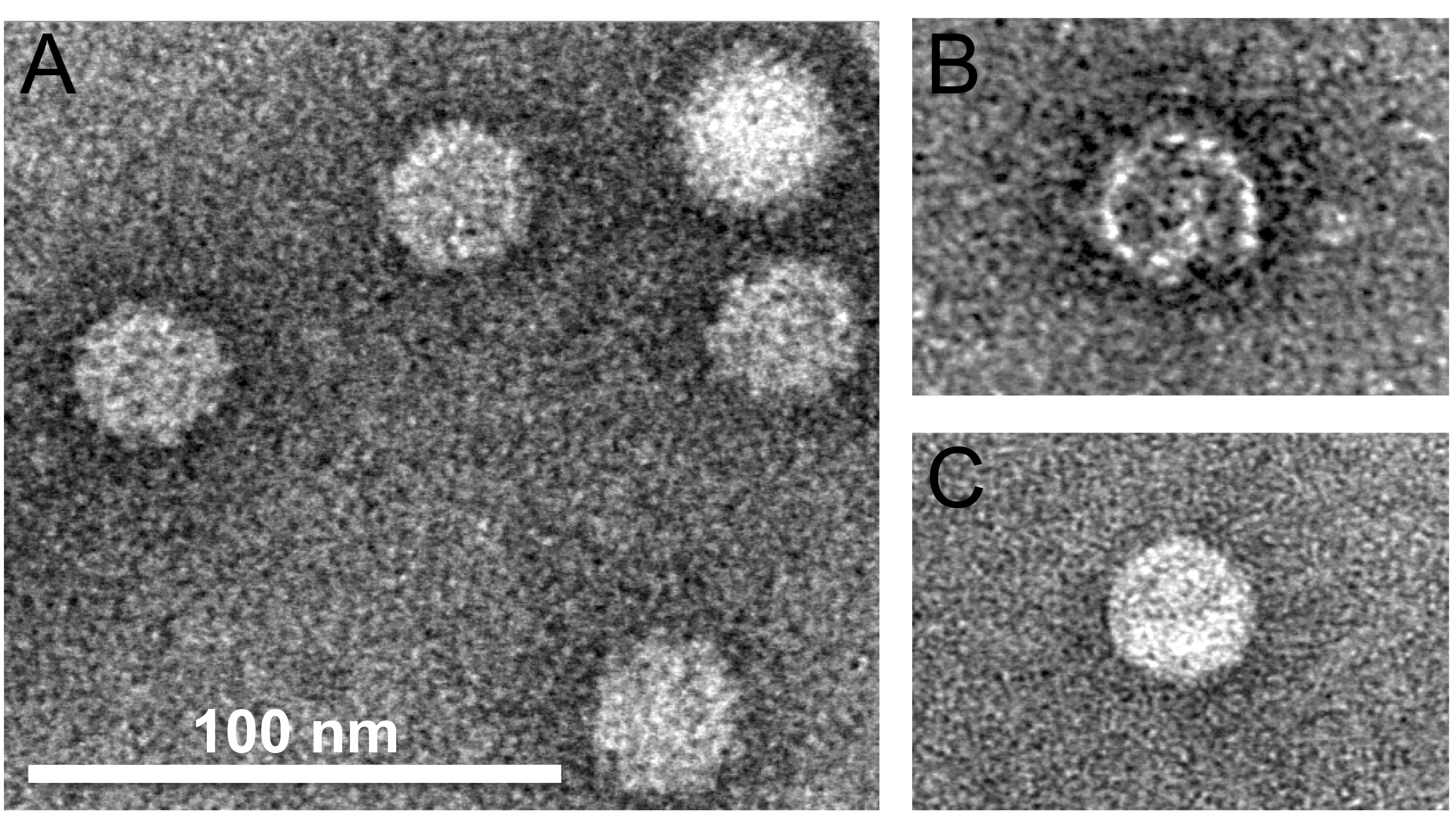

Supplement: S2 Fig — Electron micrographs of negatively stained VLPs used for crystallization. (A) T = 3 GNNV-LPs; (B) T = 1 SVPs of the N-ARM deletion mutant; (C) the delta-P-domain mutant. Bar: 100 nm. (TIFF) [file ppat.1005203.s002.tiff]

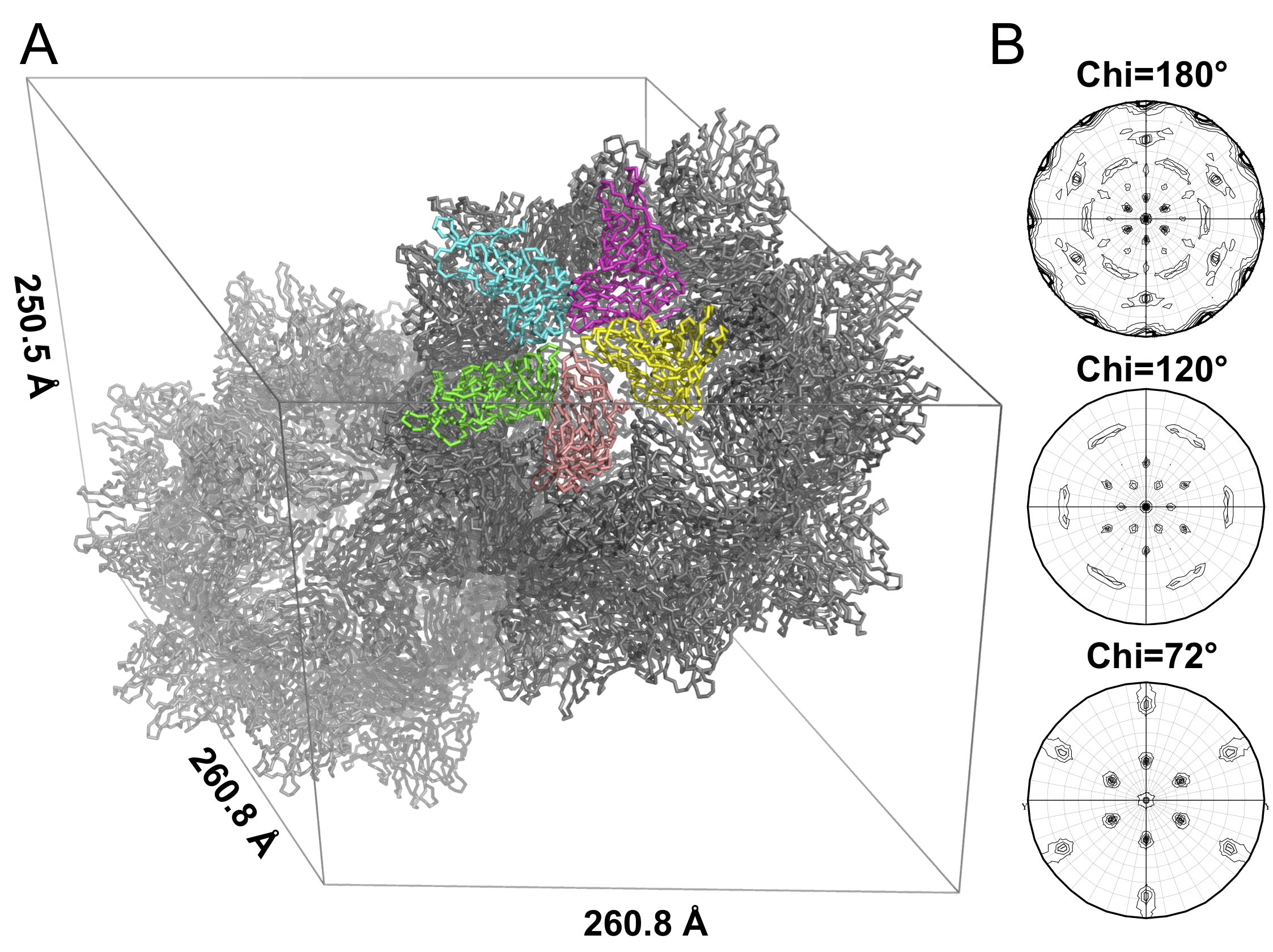

Supplement: S3 Fig — (A) Crystal packing of N-ARM deletion mutant in T = 1 assembly is well arranged in the corresponding unit cell with dimensions of a = b = 260.8 Å, c = 250.5 Å and γ = 120° in space group P6322. Five neighboring subunits of CP along the I5 axis are labeled as green, wheat, cyan, yellow and magenta colors, respectively. (B) Analyses of self-rotation functions of the T = 1 N-ARM deletion mutant. The NCS relationship was corroborated by the self-rotation functions of κ = 72°, 120° and 180° hemispheres, and calculated with Molrep [57]. (TIFF) [file ppat.1005203.s003.tiff]

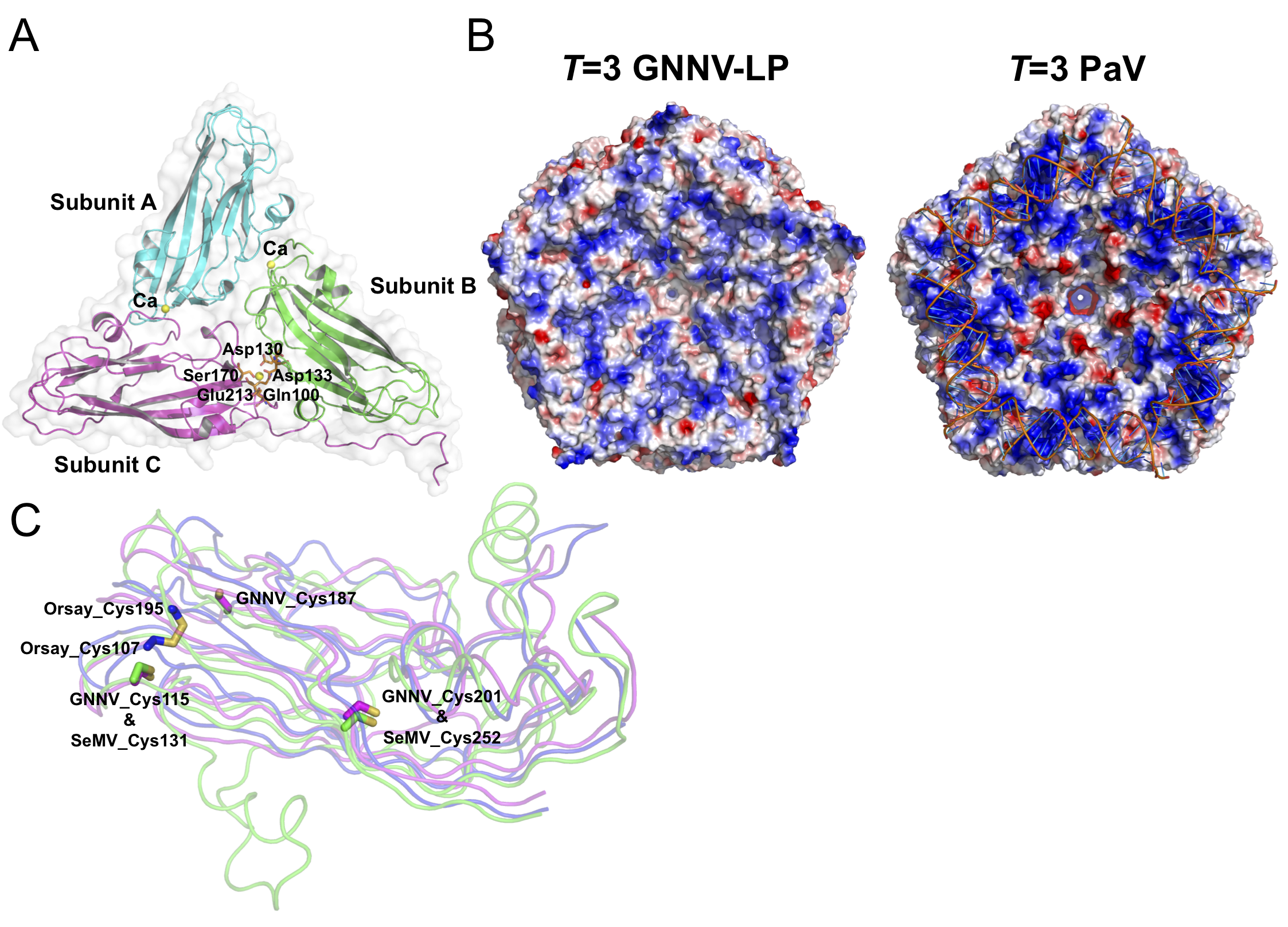

Supplement: S4 Fig — (A) S-domains of three subunits per iASU. Three Ca2+ ions (yellow spheres) are incorporated at the interfaces of neighboring subunits A (cyan), B (green) and C (magenta), where Gln100, Asp130, Asp133, Ser170 and Glu213 (orange sticks) participate in Ca2+ coordination. (B) An illustration of the geometric and electrostatic differences on the inner surface between GNNV (left) and PaV (right). The charge distribution around the inner surface of the pentameric capsomers of empty GNNV-LP and PaV has a positive electrostatic potential (blue), and five short ordered encapsidated RNA duplexes are shown along the I2 axes. (C) The distribution of cysteine residues in the S-domains. A superimposition of S-domain structures from GNNV (magenta), SeMV (green, PDB ID: 1X33) and Orsay virus (blue, PDB ID: 4NWV) is shown. Cysteine residues are shown in stick, and Cys107−Cys195 of Orsay virus represents the disulfide linkage. (TIFF) [file ppat.1005203.s004.tiff]

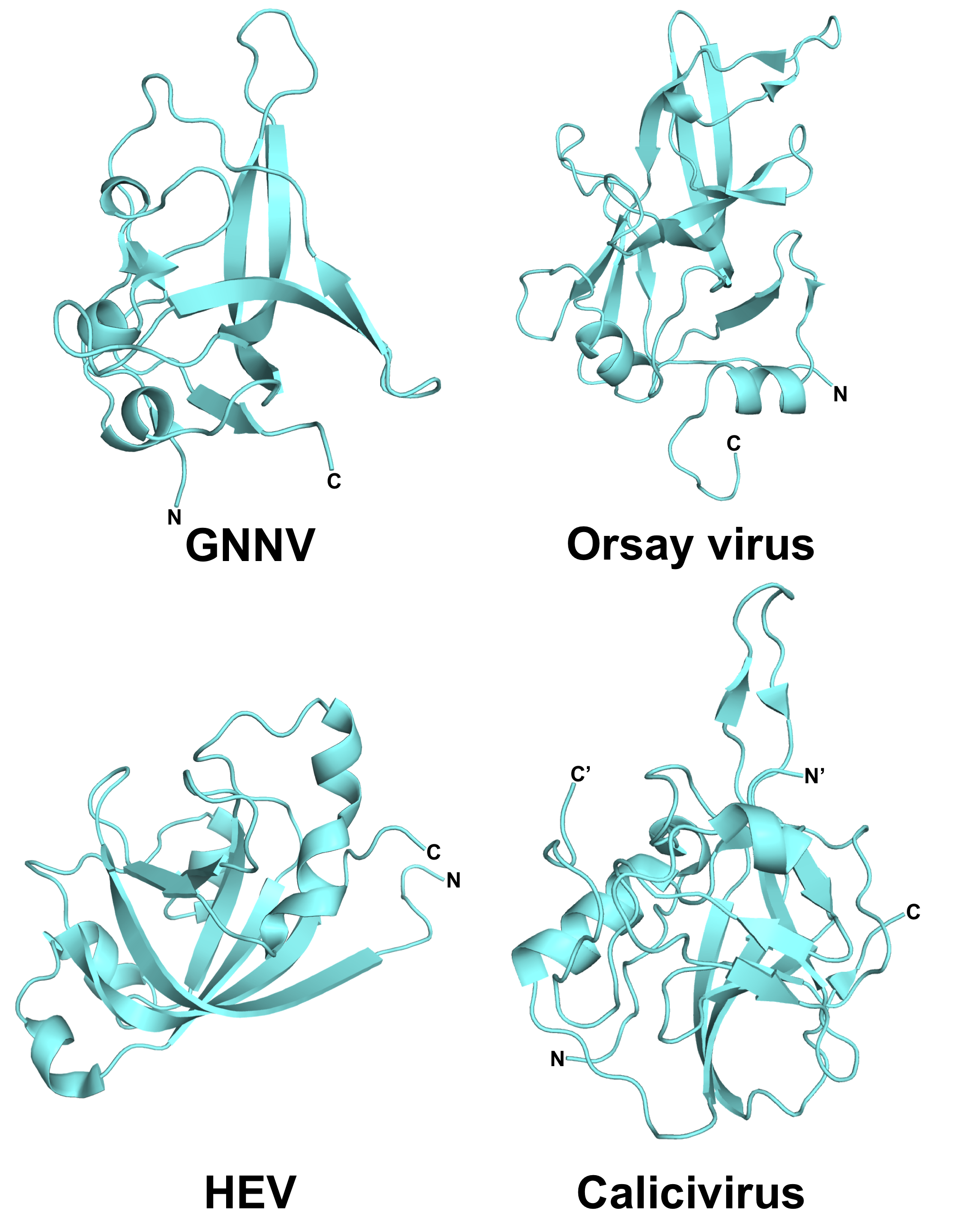

Supplement: S5 Fig — The crystal structures of the P-domain of the GNNV and Orsay viruses (PDB ID: 4NWV), and P1-domain of HEV (PDB ID: 2ZTN) and Calicivirus (PDB ID: 2GH8) are shown as ribbon diagrams. The N’ and C’ terminus of Calicivirus indicate the connecting regions between the P1 and P2 domains. (TIFF) [file ppat.1005203.s005.tiff]

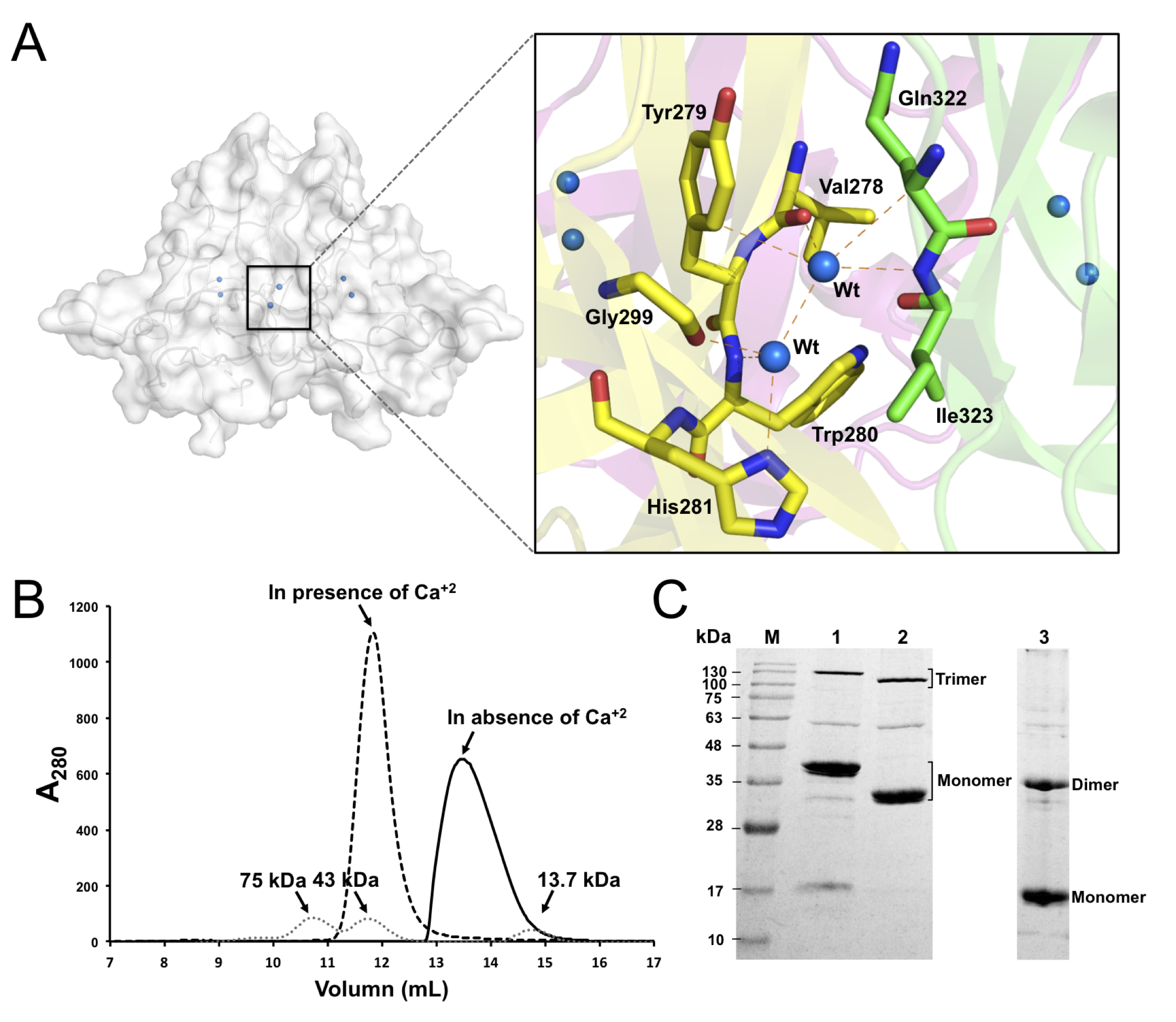

Supplement: S6 Fig — (A) Conservation of water molecules at the interfaces of neighboring subunits in the truncated P-domain. Three sets of two water molecules (blue spheres), at the subunit-​interface regions, are coordinated with the conserved residues shown from two neighboring subunits in yellow and green, respectively. (B) The equilibrium properties of the monomeric and trimeric P-domains. The purified GNNV P-domain was analyzed in the absence (solid line) or presence (dashed line) of Ca2+ using size-exclusion chromatography (SEC) on a Superdex 75 10/300 GL column (GE Healthcare). These data were compared to protein standards (conalbumin, 75 kDa; ovalbumin, 43 kDa; ribonuclease A, 13.7 kDa). (C) SDS-PAGE analysis of oligomerization of three CPs in solution. Full-length GNNV CP (lane 1) and the N-ARM deletion mutant (residues 35−338) (lane 2) exhibit monomeric and trimeric forms concurrently; and monomeric and dimeric forms of the delta-P-domain mutant (residues 35−217) (lane 3) are shown compared to protein standards (lane M). (TIFF) [file ppat.1005203.s006.tiff]

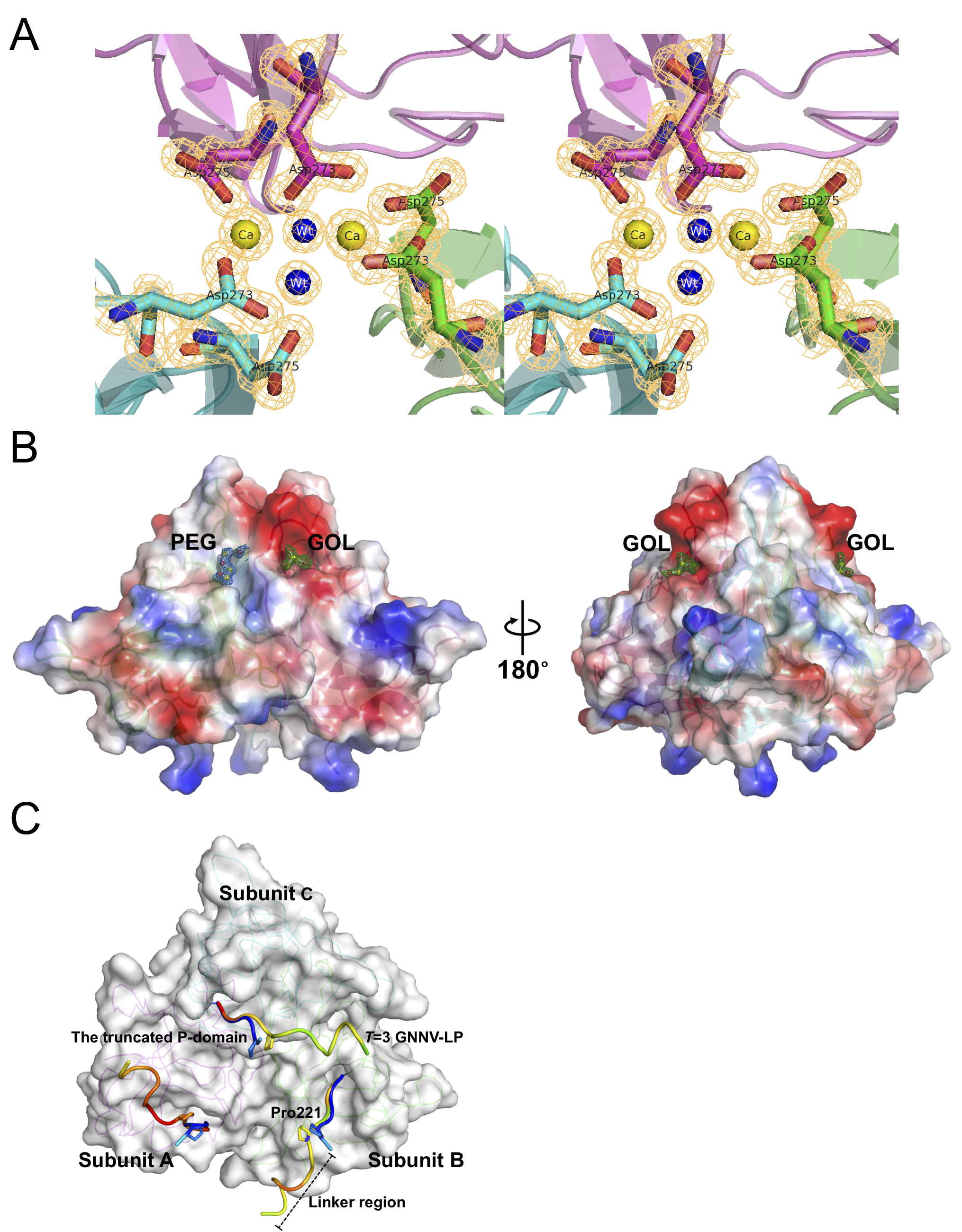

Supplement: S7 Fig — (A) A stereo view of the trimeric P-domains with Ca2+ ions and water molecules bound at the DxD motif. Two residues (Asp273 and Asp275, in sticks) coordinating two Ca2+ ions (yellow spheres) and water molecules (blue spheres) are conserved on the P-domain of each neighboring subunit. The |2F o–F c| map (orange mesh) around the binding site is shown with a contour level at 3σ. (B) There are three conserved GOL-binding pockets and one PEG-binding site on the electrostatic surface of the trimeric P-domains. Electron density maps of PEG (blue) and GOL (green) are shown with the |2F o–F c| coefficient and contour at 1σ around the interaction site. (C) A comparison of the linker regions between the truncated P-domain and the T = 3 GNNV-LP. Each hinge region is identified and color-coded as a rainbow gradient with respect to B-factor values (blue, lowest; red, highest) to depict the relatively rigid and flexible area. The three subunits (A, B and C) are shown with magenta, green and cyan, respectively. (TIFF) [file ppat.1005203.s007.tiff]

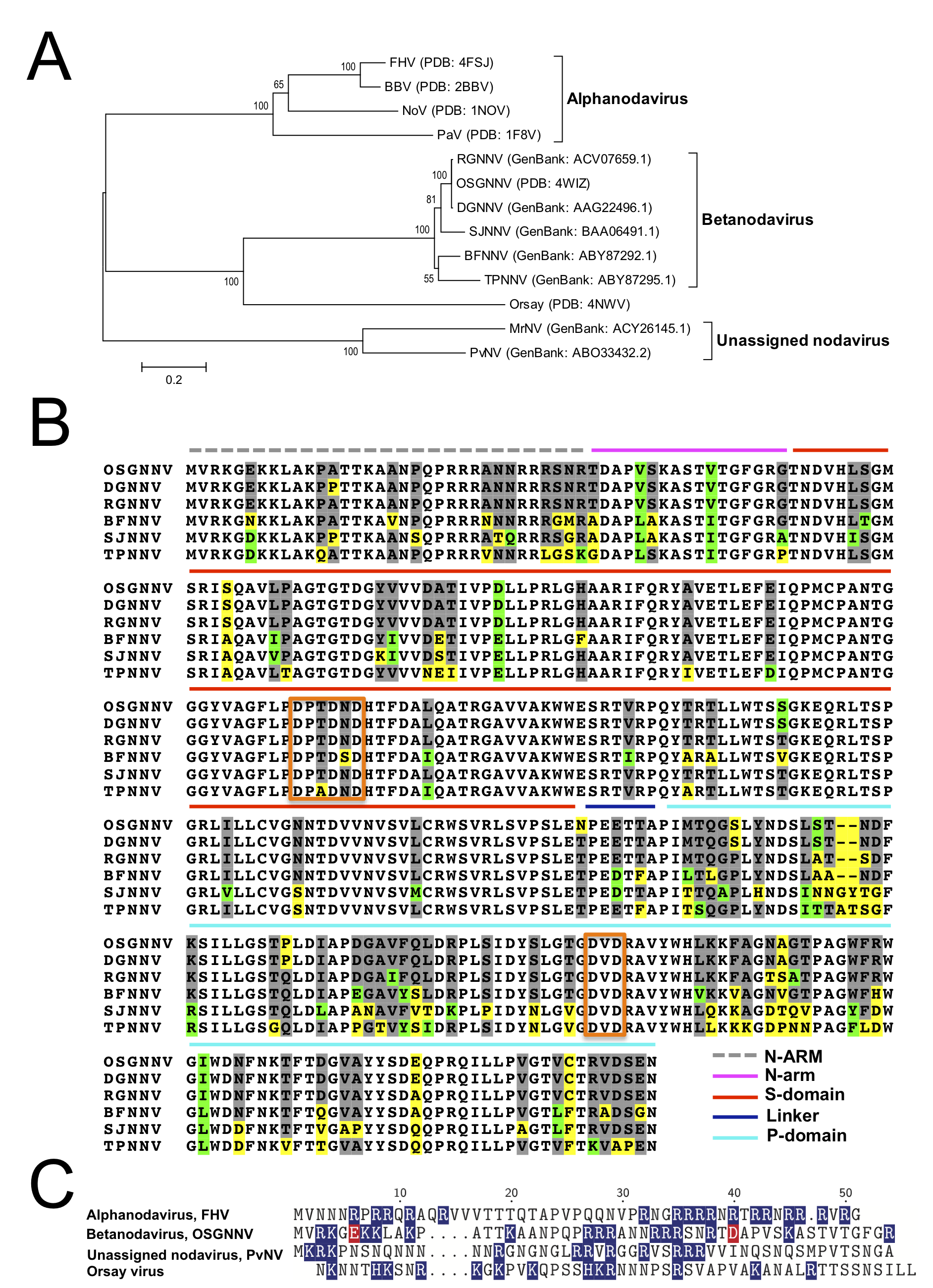

Supplement: S8 Fig — (A) The phylogenetic tree of the family Nodaviridae. The neighbor-joining phylogenetic unrooted tree was built using Mega5 [72] with a multiple alignment of conserved blocks of the sequence of RNA2. The major clades of the family Nodaviridae are identified as alphanodavirus, betanodavirus, unassigned nodavirus and Orsay virus, respectively. (B) The sequence alignment of RNA2-encoded CP from different genotypes of betanodavirus. Multiple sequence alignment was performed with sequences of the CPs from OSGNNV, DGNNV, RGNNV, BFNNV, SJNNV and TPNNV using ClustalW. Each domain of GNNV CP is indicated on the top of alignment with colors as in Fig 1B. The DxxDxD and DxD motifs of GNNV CP are identified in the orange boxes. (C) N-terminal sequence identification of the CPs from different strains of the family Nodaviridae. Basic- and acidic-charged residues are colored in blue and red, respectively. (TIFF) [file ppat.1005203.s008.tiff]
